# Characterization of MdMYB68, a suberin master regulator in russeted apples

**DOI:** 10.3389/fpls.2023.1143961

**Published:** 2023-03-20

**Authors:** Xuan Xu, Gea Guerriero, Frederic Domergue, Olga Beine-Golovchuk, Emmanuelle Cocco, Roberto Berni, Kjell Sergeant, Jean-Francois Hausman, Sylvain Legay

**Affiliations:** ^1^ Environmental Research and Innovation (ERIN) Department, Luxembourg Institute of Science and Technology, Esch-Sur-Alzette, Luxembourg; ^2^ Université de Bordeaux, Centre National de la Recherche Scientifique (CNRS) – Unité Mixte de Recherche (UMR) 5200, Laboratoire de biogenèse Membranaire, Bâtiment A3 ‐ Institut Natitonal de la Recherche Agronomique (INRA) Bordeaux Aquitaine, Villenave d’Ornon, France

**Keywords:** russeting, suberin, cell wall, *Malus* x *domestica*, *Nicotiana benthamiana*, MYB-family transcription factor, MdMYB68, Transcriptomics

## Abstract

**Introduction:**

Apple russeting is mainly due to the accumulation of suberin in the cell wall in response to defects and damages in the cuticle layer. Over the last decades, massive efforts have been done to better understand the complex interplay between pathways involved in the suberization process in model plants. However, the regulation mechanisms which orchestrate this complex process are still under investigation. Our previous studies highlighted a number of transcription factor candidates from the Myeloblastosis (MYB) transcription factor family which might regulate suberization in russeted or suberized apple fruit skin. Among these, we identified *MdMYB68*, which was co-expressed with number of well-known key suberin biosynthesis genes.

**Method:**

To validate the MdMYB68 function, we conducted an heterologous transient expression in Nicotiana benthamiana combined with whole gene expression profiling analysis (RNA-Seq), quantification of lipids and cell wall monosaccharides, and microscopy.

**Results:**

MdMYB68 overexpression is able to trigger the expression of the whole suberin biosynthesis pathway. The lipid content analysis confirmed that MdMYB68 regulates the deposition of suberin in cell walls. Furthermore, we also investigated the alteration of the non-lipid cell wall components and showed that MdMYB68 triggers a massive modification of hemicelluloses and pectins. These results were finally supported by the microscopy.

**Discussion:**

Once again, we demonstrated that the heterologous transient expression in *N. benthamiana* coupled with RNA-seq is a powerful and efficient tool to investigate the function of suberin related transcription factors. Here, we suggest MdMYB68 as a new regulator of the aliphatic and aromatic suberin deposition in apple fruit, and further describe, for the first time, rearrangements occurring in the carbohydrate cell wall matrix, preparing this suberin deposition.

## Introduction

Apple russeting is a rough and brown phenotype which results from defects or damages on the fruit surface leading to price downgrading and loss of profitability for producers (Faust and Shear, 1972; Skene, 1981). Russesting is caused by *de novo* suberin deposition, also called suberization, in epidermal cells of apple fruits (Faust and Shear, 1972; Legay et al., 2017). Suberin itself is a cell wall polymer which comprises two distinct aromatic and aliphatic domains forming an hydrophobic protective barrier against environmental stresses at the fruit surface (Skene, 1981; Woolfson et al., 2022).

Russeting can be triggered by multiple causes including cold, wet weather, pesticides and pathogen attacks (Faust and Shear, 1972), and mechanisms involved in suberin deposition have been deeply unraveled this last decade. Several transcriptomic studies revealed that apple russeting involved a complex interplay between multiple metabolic pathways such as hormone signaling and phenylpropanoid and lipid metabolisms (Lashbrooke et al., 2015; Legay et al., 2015; Falginella et al., 2021; André et al., 2022). A numerous suberin biosynthesis genes have already been described in model plants and are covering the whole suberin biosynthetic pathway including the specific synthesis of hydroxycinnamic acids such as ferulic acid, or of suberin specific very long chain fatty acids, fatty alcohols, ω-hydroxyacids and α,ω-dicarboxylic acids (for reviews, please see Bernards (2002); Graça (2015); Vishwanath et al. (2015); Serra and Geldner (2022); Woolfson et al. (2022)). In apple, our studies also highlighted an important alteration of the expression of genes involved in cell wall modification during the suberization of fruit periderms. In particular, the expression of genes associated with hemicellulose (xylan and xyloglucans) and pectin modifications was affected in a similar way to what has been previously associated with cell wall loosening (Legay et al., 2015). However, the alteration of cell wall components in suberizing tissues has been poorly studied and still needs to be validated.

In addition to biosynthesis genes,.transcriptomic datasets of the suberin deposition highlighted numerous genes coding for Myeloblastosis protein (MYB) and No Apical Meristem (NAC) domain transcriptional regulators, which constituted promising suberin biosynthesis regulator candidates. The first suberin biosynthesis transcription factor (TF, AtMYB41) was discovered in *Arabidopsis thaliana* and described as a trigger of suberin deposition in response to abscisic acid (ABA) and NaCl stress (Kosma et al., 2014). In apple, the orthologous gene of AtMYB41 was not expressed in russeted skin, suggesting that other TFs might be implicated in the regulation of suberin deposition during russeting (Legay et al., 2015; André et al., 2022). MdMYB93 was the first transcriptional regulator associated with the suberin deposition in apple fruit skin (Legay et al., 2016). In *Nicotiana benthamiana* leaves, the transient expression of apple *MdMYB93* triggered a massive increased expression of genes belonging to the phenylpropanoid and lipid metabolisms with a concomitant deposition of suberin in cell walls. Recently, we described MdMYB52, which is specifically expressed in russeted tissues and regulates the biosynthesis of G-units lignin associated with the aromatic domain of suberin (Xu et al., 2022b). Other members of the MYB family were never studied in apple fruit although several other suberin transcriptional regulators were recently discovered in *A. thaliana*, including MYB9, MYB36 MYB107, MYB53, MYB92 and MYB39/SUBERMAN to name a few (Kamiya et al., 2015; Lashbrooke et al., 2016; Cohen et al., 2020; To et al., 2020; Shukla et al., 2021). According to whole gene expression studies performed on apple suberizing tissues, some of these transcription factors might also regulate the suberin deposition in apple fruit skin, but further functional studies are required to validate this hypothesis (Falginella et al., 2021; André et al., 2022).

Interestingly, a recent study proposed for the first time, a model aiming to explain an upstream/downstream regulation pathway for suberin biosynthesis, describing tier levels and TF-TF interactions (Xu et al., 2022a). This model highlighted that the Arabidopsis orthologous gene of MdMYB93 is in the downstream part of the regulatory cascade, whereas a AtMYB68 seemed to be involved in the upstream tier. Interestingly, our previous transcriptomic datasets demonstrate an increased expression of the apple orthologous gene MdMYB68. In the present study, we further investigated the possible involvement of MdMYB68 in the suberization process in apple fruit skin using transient expression in *N. benthamiana* associated with a whole gene expression profiling using RNA-Seq, lipid and cell wall content analysis, and finally microscopy.

## Materials & methods

### RNA extraction, reverse transcription, and qPCR

For the apple *MdMYB68* (MD08G1076200) gene isolation, total RNA were obtained from excised fruit peel of the semi-russeted *Malus* x *domestica* cv. “Cox Orange Pippin” variety using an adapted CTAB buffer-based extraction protocol (Gasic et al., 2004) and further used to generate cDNA backbone and gene specific amplification. For the gene expression, *N. benthamiana* RNA were extracted from two groups, (p103:: MD08G1076200/pBIN61-p19 (overexpression) and p103 (empty vector)/pBIN61-p19 (control)), made of 4 biological replicates constituted themself by 3 plants and 4 infiltrated leaves each. Total RNA extracts were obtained using the RNeasy plant mini kit (QIAGEN, Leusden, The Netherlands) coupled with on-column DNase I treatment, following the manufacturer’s guidelines. Total RNA integrity (RIN>8) and purity were assessed using a 2100 Bioanalyzer (Agilent Technologies, Santa Clara, CA, USA) and a Nanodrop ND1000 spectrophotometer (Thermo scientific, Villebon-sur-Yvette, France), respectively. RNA concentration was quantified using a Qubit RNA assay kit (Life technologies, Carlsbad, CA, USA). For gene isolation and RT-qPCR, reverse transcription was carried out as described in our previous work and primers details are available in **Supplementary Table 1** (Legay et al., 2015).

### MdMYB68 gene isolation and cloning

To achieve *MdMYB68* amplification, a total RNA from *Malus* x *domestica* cv. “Cox Orange Pippin” has been extracted and corresponding cDNA backbone was generated. The reverse transcription was carried out using the M-MuLV Reverse Transcriptase (RNase H-), the Murine RNase Inhibitor (New England Biolabs, Ipswich, MA, USA) and random hexamers (Invitrogen, Carlsbad, NM, USA) following the manufacturer’s guidelines. The *MD08G1076200* cloning was obtained using a PCR amplification (Q5 High Fidelity 2X Master Mix (New England Biolabs, Ipswich, MA, USA) following the manufacturer’s instructions (Tm=65°C). The specific primers: Forward(F) 5’- CACCATGGAGGATTATGGGGATGA-3’ and Reverse(R) 5’- AGAAGAGATCCCCACACCAA -3’ were used to generate amplicon. The PCR product was purified using the QIAquick PCR purification Kit (QIAGEN), cloned into pENTR/D TOPO and further used to transform *E. coli* One Shot TOP10 competent cells, which were grown on LB/kanamycin (50 mg/L) plate according to the manufacturer’s guidelines (Thermo scientific). The colony with containing plasmid was then selected and plasmid was extracted using the QIAprep Spin Miniprep Kit (QIAGEN, Leusden, The Netherlands). The insert was sequenced applying gene specific primers (LGC genomic service Berlin, Germany). The LR clonase cloning protocol (Thermo scientific) was used to recombine the insert into pEarleyGate103(p103) vector (Earley et al., 2006) to obtain a MD08G1076200-GFP fusion driven by the CaMV35S promoter. After quality check using sequencing P103:: MD08G1076200 was finally transformed into *Agrobacterium tumefaciens* GV3101-pMP90.

### Infiltration by *Agrobacterium tumefaciens*

*A. tumefaciens* GV3101-pMP90 strains (p103::MD08G1076200, p103-empty and pBIN61-p19) were grown in 50 mL of LB liquid medium supplemented with gentamycin (30 mg/L), rifampicin (10 mg/L), kanamycin (50 mg/L), and acetosyringone (30 mg/L). As *A. tumefaciens* is not sensitive to the CcdB protein, we used the p103-empty vector as control in the present work (Traore and Zhao, 2011). Cultures in 100 mL Erlenmeyer flasks were agitated at 130 rpm/30°C for 48 h. After centrifugation (1000 g/10 min), cultures were re-suspended in infiltration buffer (20 mM MES, 20 mM MgSO_4,_ 150 mg/L acetosyringone). After 3 hours, p103::MD08G1076200, p103-empty and pBIN61-p19 *A. tumefaciens* cultures were mixed and adjusted to OD_600 =_ 0.8, 0.8 and 1, respectively. For the RNA-Seq, qPCR validation, cell wall analysis and lipid analysis, independent transient expression experiments have been performed using p103:: MD08G1076200/pBIN61-p19 and p103-empty vector/pBIN61-P19 A. tumefaciens mixtures using four biological replicates (n=4). Each biological replicate is constituted by three plants and four leaves per plants. Leaf samples were collected (i) 4 days after infiltration for gene expression experiments (RT-qPCR and RNA-Seq), (ii) 7 days after infiltration for the cell wall analysis, lipid analysis and microscopy experiments.

### cDNA library preparation and sequencing

cDNA libraries were prepared from 3 µg total RNA of each biological replicate, collected 4 days after infiltration, according to the KAPA HyperPrep Kit manufacturer’s guidelines (Roche, Basel, Switzerland). All libraries were analyzed and quantified as described in (Legay et al., 2016). The sequencing was carried out at the LCSB sequencing platform (RRID: SCR_021931) at the University of Luxembourg. The pooled libraries were sequenced on an Illumina NextSeq500 (NextSeq 500/550 Mid Output Kit v2.5 (150 Cycles)) to generate 75 base-pairs paired-end reads. FASTQ files were imported in pairs in CLC genomics workbench v11.0.1 discarding poor-quality reads (<Q30). For each library, reads were trimmed and filtered using the following criteria: sequence quality score <0.01, no ambiguous nucleotides, minimum read length >35 nucleotides, trimming against the Illumina adaptor sequence, and finally a hard trim of 14 nucleotides at the 5’ end and 3 nucleotides at the 3’ end. The filtered reads were mapped to the *N. benthamiana* transcriptome v5-primary and alternate transcript (Nakasugi et al., 2014) obtained from the *N. benthamiana* genome and transcriptome website (http://benthgenome.qut.edu.au/) with the following criteria: a mismatch, gap and insertion cost set at 2,3 and 3 respectively (3=stringent mapping), reads should have 80% identity and 80% coverage with the reference transcriptome.

### Gene expression and gene ontology calculations

Expression values were calculated using the RPKM (Reads per kilobase transcript per million reads) method (Mortazavi et al., 2008). To determine the differentially expressed genes between the tobacco leaves infiltrated with p103:: MD08G1076200/pBIN61-p19 and p103-empty vector/pBIN61-P19, a Baggerley’s ‘on proportions’ weighted test (Baggerly et al., 2003) combined with a false discovery rate correction (Benjamini-Hotchberg) set at 0.001 was used. Expression cut-off values were set at 4-fold increase/decrease and 5 RPKM difference, respectively. The corresponding gene identifiers were uploaded into the bioinformatics g:Profile web server https://biit.cs.ut.ee/gprofiler/ and mapped as source for gene ontology enrichment analysis (Raudvere at. al. 2019). To highlight the most significantly regulated ontology groups the two GOs outlier with the strongest p-value were selected and uploaded into the Cytoscape (v3.6.0) web server. Based on this, an interaction network applying the ClueGO v2.5 and CluePedia v1.5 plugins (Bindea et al., 2009) was built and the most regulated biological processes were highlighted. Raw sequences have been deposited at the NCBI Gene Expression Omnibus website (GEO, http://www.ncbi.nlm.nih.gov/geo, accession number: GSE220694).

### Monosaccharide quantification from total and fractionated cell wall materials

The extraction of cell wall materials was performed from samples collected 7 days after infiltration as described previously (Xu et al., 2019; Guerriero et al., 2021). Leaves infiltrated with the empty vector and the TF (four biological replicates per treatment) were lyophilized and subsequently ground to a fine powder. Fifty mg of the ground material were extracted on ice three times with 80% (v/v) ethanol for 30 min, followed by two washes, the first with acetone then with methanol at RT. One hundred U of α-amylase (porcine pancreas, Sigma-Aldrich, Merck, Darmstadt, Germany) were added to the residues at 40°C during an incubation time of 1 h, then 50 U α-amylase were further added for 30 min to completely remove starch. The cell wall materials were precipitated by four volumes of cold ethanol (100% v/v) o/n, followed by three washes with cold ethanol. After air-drying, five mg of cell wall materials were hydrolyzed using one-step two-step hydrolysis, i.e., Saeman’s hydrolysis for the swelling of cellulose, followed by matrix polysaccharide hydrolysis with diluted acid (72% w/w sulfuric acid, vortexed intermittently at RT for 1h, then diluted to 10% w/w and incubated for 3 h at 100°C) (Yeats et al., 2016). The samples were thereafter cooled at RT and centrifuged. The supernatant was used for the determination of the cell wall monosaccharide composition

For the cell wall fractionation protocol, 15 mg of air-dried cell wall materials were subjected to sequential extraction using water, 0.1% (w/v) EDTA (pH 7.5), 1 M and 4 M KOH. The extractions with water and EDTA were performed for a total of three times at 99°C for 2 h each. Alkaline extractions with KOH were carried out with 0.01 mM NaBH_4_ at RT for 2 h, then, glacial acetic acid was used to neutralize the extracts. Dialysis of all the extracts was carried out against deionized water and concentrated by rotary evaporation. The residues after sequential extraction were rinsed three times with deionized water. All fractions and residues were freeze‐dried. The cell wall monosaccharide composition was determined with high-performance ion chromatography (in technical duplicates for each sample) using a Dionex™ ICS-5000+ Capillary HPIC™ System (Dionex, Thermo Fisher Scientific, Bremen, Germany), as previously reported (Xu et al., 2019).

### Soluble and polymerized lipid fraction analysis

*N. benthamiana* leaves infiltrated on the abaxial side of the leaves with *A. tumefaciens* strains (p103::MD08G1076200/pBIN61-p19 and p103-empty vector/pBIN61-P19, n=4), were harvested after 7 days, lyophilized and stored at 4°C until further processing. Delipidation steps, and suberin composition and content analysis were performed using the solvent-extraction method as described in Delude et al. (2017).

### Microsocopy

Seven days after infiltration, *Nicotiana benthamiana* leaf samples were collected and washed with phosphate buffer twice for 10 min, then dehydrated in a graded series of ethanol and resin embedded as described previously (Xu et al., 2022a). For immuno‐localization, sections (10µm) of tobacco leaves were processed as described in (Behr et al., 2016). Sections were incubated for 1.5 h at room temperature with the primary antibodies LM1, LM2, LM5, LM10 and INRA-RU1 diluted 1:10 in the same buffer. Sections were washed for 20 min in the dilution buffer + 0.1% (v/v) Tween20 and incubated for 45 min at room temperature with secondary antibody diluted 1:100 in 0.02 M Tris‐HCl pH 8.2 (Behr et al., 2016; Xu et al., 2019). All antibodies were purchased from Plant-Probes (http://www.plant.probes.net) excepted for INRA-RU1, which was kindly provided by Dr. Marie-Christine Ralet (INRA Angers-Nantes). For Fluorol yellow 088 staining, sections were stained with a freshly prepared solution of Fluorol Yellow 088 (0.1% w/v/v/v, in PEG 400/glycerol/water) at 70°C for 30 min. Sections were rinsed with water (three rinses of 5 min each). A counter-staining was done with Aniline Blue (0.5% w/v, in water) at room temperature for 30 min in darkness. Samples were washed with water (three rinses of 5 min each). Samples were observed with an Olympus microscopy BX43 Fluo. DAPI filter was selected. The UV intensity was regulated through a LED illumination unit (pE-300 white).

## Results

### MdMYB68 is co-expressed with typical suberin biosynthesis genes

**Figure 1:**
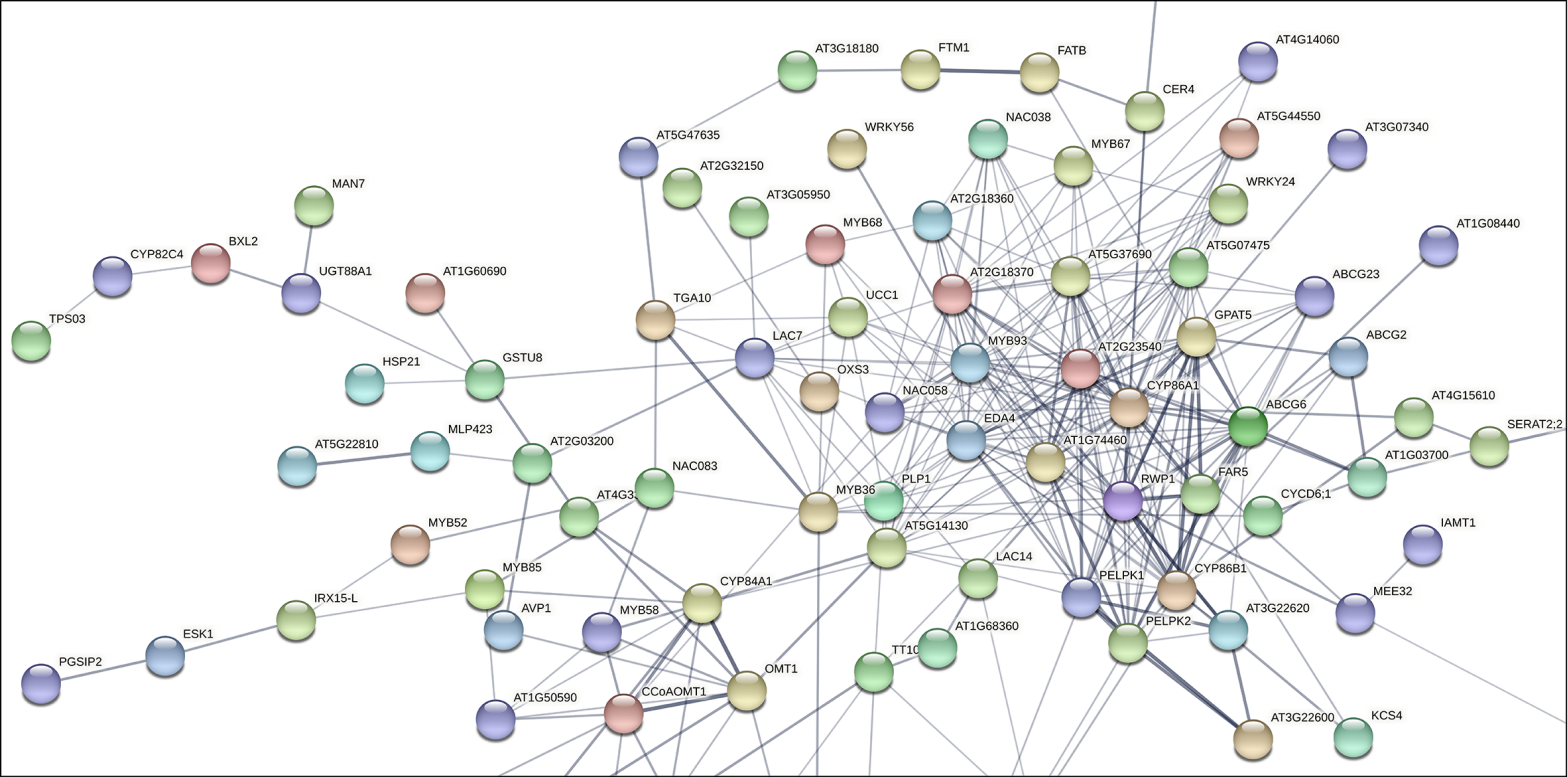
Subset of the gene interaction matrix obtained from genes significantly upregulated in russeted apple fruit skin compared to waxy apples (-3<log_2_ ratio (russeted/waxy)<3) (Legay et al., 2015; André et al., 2022). Grey lines represent the gene interaction confidence (ranged from 0 to 1): thickest lines display confidence scores higher than 0.9, thinnest lines display confidence scores between 0.4 and 0.7. Node color display the different shell of interactors (from hot colors for the first shells of interactors to cold colors for the last shells). The complete gene interaction map, gene description and associated gene ontology analysis are available in **Supplementary Figure 1** and **Supplementary Table 2**.

To assess the putative involvement of MD08G1076200 (MdMYB68) as a transcriptional regulator of the suberization process in russeted apple skin, a gene interaction matrix was first built from significantly upregulated genes observed in previous transcriptomic datasets comparing gene expression profiles in russeted vs waxy apple skin tissues (Legay et al., 2015; André et al., 2022). The interaction mapping clearly highlighted the lipid and phenylpropanoid main bioprocess clusters (**Figure 1**, **Supplementary Figure 1**, **Supplementary Table 2**).

As expected, the lipid metabolism cluster included genes involved in the biosynthesis of the suberin aliphatic domain of suberin i.e. the cytochrome P450 family 86 subfamily A1 (*CYP86A1*), cytochrome P450 family 86 subfamily B1 (*CYP86B1*), glycerol-3-phosphate acyltransferase 5 (*GPAT5*), fatty acid reductase 5 (*FAR5*), ω-hydroxyacid-O-hydroxycinnamoyl transferase (*RWP1*), MYB93 and multiple GDSL-lipase/esterase to name a few (Vishwanath et al., 2015; Legay et al., 2016; Woolfson et al., 2022). MD08G1076200/MdMYB68 displayed weak interaction (0.150 < confidence < 0.400) with several proteins of this cluster including: (i) the Bzip transcription factor familly protein TGA10 involved in the anther development; (ii) MYB36 involved in the regulation of the casparian strips formation in Arabidopsis endodermal cells (Kamiya et al., 2015); (iii) Embryo sac development 4 (EDA4) a lipid transfer protein; (iv) a gene similar to Ucalacyanin 1 (UCC1) involved in the deposition of the poly(phenolic) deposition in Casparian strips (Woolfson et al., 2022); (v) a GDSL-lipase/esterase similar to GELP38/AT1G74460 which is required for the final formation of the suberin poly(alipihatic) domain (Ursache et al., 2021) and finally (vi) an α/β-hydrolase (AT2G18360) for which no clear function has been proposed yet (**Figure 1**). Altogether, this co-expression matrix suggests that MdMYB68 is another important component of the regulation of the suberin biosynthesis in russeted apple fruit skin. Interestingly, this interaction mapping also highlighted cell wall metabolism as a major bioprocess regulated by MD08G1076200/MdMYB68.

### MdMYB68 transient expression strongly altered the expression of genes involved in the different steps of suberin deposition

To justify the function of MdMYB68 in suberin biogenesis and deposition in apple russeted skin, we performed a transient heterologous expression of MdMYB68 in *N. benthamiana* leaves. CDNA libraries were prepared from mRNA extracted from infiltrated leaves to perform a whole-gene expression profiling analysis as previously described in Kosma et al. (2014) and Legay et al. (2016). Our data collected about 29 to 34 million of mapped reads per libraries corresponding to about 84 to 88% mapping rate against the *N. benthamiana* transcriptome (**Supplementary Table 3**). To calculate gene expression rate between mock and MdMYB68 overexpression, we used RPKM methodology (Reads per kilobase transcript per million reads). 3305 expressed genes were selected, based on their significances (Benjamini-Hochberg FDR corrected p-value <0.001). Around 86% (a set of 2840 genes) were classified as highly upregulated genes (**Supplementary Table 4**). Expression of a subset of genes was also analyzed using RT-qPCR and displayed a strong correlation with RNA-Seq data (**Supplementary Table 1**).

**Figure 2:**
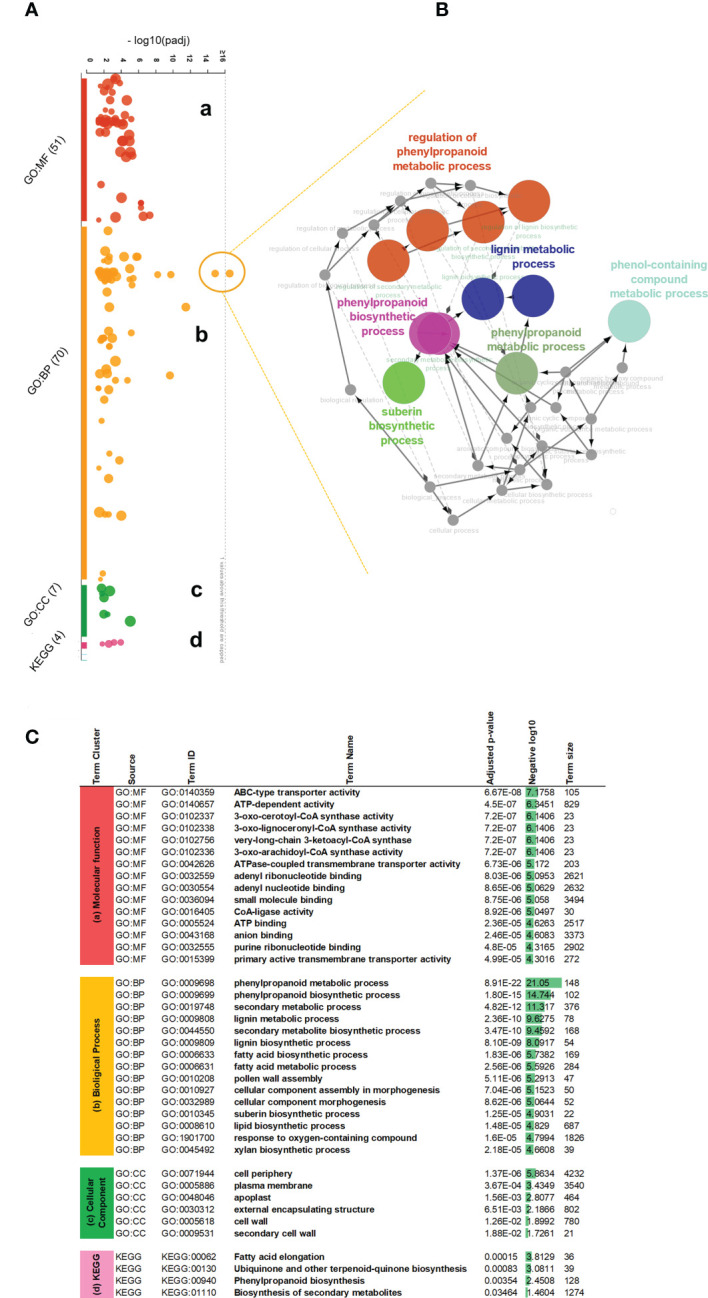
Gene ontology enrichment analysis of the differentially expressed genes obtained 4 days after agroinfiltration with empty vector and MdMYB68 constructs in *N. benthamiana*. **(A)** An interactive Manhattan plot visualizes enriched GO term cluster within plant response to MYB68 overexpression. The x-axis shows the functional terms from 2840 upregulated genes. The circle on the plot represents a single functional term. Each circle is color-coded and grouped into the functional terms according to the annotated genes in each term. Four main gene ontology groups are identified: (a) molecular biology, (b) biological process, (c) cellular compartment, (d) KEGG Kyoto Encyclopedia of Genes and Genomes annotation. The corresponding enrichment p-values in negative log_10_ scale are illustrated on the y-axis. The hypergeometric p-value is calculated with the g:Profiler software and represent the significance of functional term in the input gene list. **(B)** Expanded view and network mapping of the genes corresponding to GO:0009698 and GO:0009699 terms, indicating the highest p-value. The nodes are the functional groups and edges are interaction. The gene network was plotted by applying the cytoscape ClueGO v2.5, CluePedia v1.5 plugins and highlights interaction of most regulated biological processes revealed significant stimulating in the phenylpropanoid fatty acid pathway. **(C)** Table of most significant gene ontology terms enriched after MYB68 overexpression. Based on the p-value, top 15 gene terms from the cluster a (MF) molecular function and cluster b (BP) biological process, along with all terms from the cluster c (CC) cell compartments and cluster d (KEGG) were highlighted and presented as most relevant processes to be consider.

To better explain their identity within functional processes, we used corresponding Arabidopsis orthologous gene IDs and performed the functional interpretation applying gProfiler enrichment analysis. We visualized identified gene term cluster on the interactive Manhattan plot (**Figure 2A**, **Supplementary Table 5**) based on their p-value. In general, 117 GOs were found, 43 terms were clustered into the molecular function (MF), 64 terms belonged to the biological process (BP), another 6 terms associated with the cellular compartment (CC), and the last 4 terms groups matched as cluster (KEGG) meaning functional annotations from Kyoto Encyclopedia of Genes and Genomes. Among these, critical GO terms were found (**Figure 2C**): very-long-chain 3-ketoacyl-CoA synthase activity (GO:0102756), lipase activity (GO:0016298), glucuronosyltransferase activity (GO:0015020), oxidoreductase activity (GO:0016705), phenylpropanoid metabolic process (GO:0009698), lignin metabolic process (GO:0009808), fatty acid metabolic process (GO:0006631), suberin biosynthetic process (GO:0010345), xylan biosynthetic process (GO:0045492), cell periphery (GO:0071944), apoplast (GO:0048046), and cell wall (GO:0005618). This observation strongly suggested the involvement of MdMYB68 in the regulation of all the successive steps of the suberization process as it altered the expression of gene involved in the cell wall modification, the phenylpropanoid/lignin metabolism and the lipid metabolism. Moreover, this signature is highly similar to that observed in our previous work on the suberin master regulator MdMYB93 as well as our previous transcriptomic work performed in russeted apples (Legay et al., 2015; Legay et al., 2016; Falginella et al., 2021; André et al., 2022).To understand the precise functional gene interaction following MdMYB68 overexpression, we further selected two most significant gene terms GO:0009698 and GO:0009699 as an outlier-nods from the biological process and extracted the corresponding genes to perform pathway network analysis. All of these core genes encoded important enzymes which mostly contribute to the synthesis of suberin building blocks including long- and very long chain ω-hydroxyacids and α,ω-dicarboxylic acids (DCAs), or phenypropanoids such hydroxycinamic acids. Our Cytoscape analysis (ClueGO v2.5 and CluePedia) was constructed base on these 35 genes including *CYP86A1*, *CYP86B1*, *KCS2*, *KCS4*, *LACs*, *PAL*, *PAL2*, *4CL*, *HCT*, *GPAT5*. In this context, the main nod for the interaction network was mapped for phenylpropanoid biosynthesis which was closely associated with either lignin metabolic process or suberin biosynthesis (**Figure 2B**; **Supplementary Table 5**).

### MdMYB68 associates the expression of key cell wall modification and suberin biosynthesis genes

From the narrow view to the GO annotations (**Figure 2C**), we identified that the transient expression of MdMYB68 also altered the expression of gene clusters which are associated with cell wall modification. Furthermore, it is noteworthy that most of these genes were associated with the modification of hemicellulose and pectins, and might play a role in the suberization process (Legay et al., 2015; André et al., 2022). Several induced beta-1,4-xylosyltransferase (*GUT1*, *IRX14L*, *IRX9*) genes are involved in the synthesis and elongation of the glucuronoxylan xylosyl backbone (**Table 1**, Wu et al., 2010), PGSIP1-GUX1 and PGSIP3-GUX2 were shown to catalyze the addition of glucuronic acid to xylan in Arabidopsis (Rennie et al., 2012). Two mannan endo-1,4-beta-mannosidase were also upregulated and are throught to be involved, together with the xyloglucan endotransglucosylase/hydrolases (XTHs), in hemicellulose remodeling during multiple growth processes such as senescence or seed germination (Schröder et al., 2009). Our data additionally suggests that MdMYB68 triggers a remodeling of pectins as several genes including pectate lyase, polygalacturonase or rhamnogalacturonate lyase displayed a significant increase in expression. Apart from genes associated with hemicellulose and pectin, callose synthase (*GSL07*) and cellulose synthase-like D3 (*CSLD3*) were upregulated. Finally, we observed a large number of genes coding for extensins (Mishler-Elmore et al., 2021).

**Table 1:** Selection of the differentially expressed genes obtained from the statistical comparison of *N. benthamiana* leaves infiltrated with the MYB68 construct versus leaves infiltration with empty vector construct 4 days after infiltration.

Contig Name	RPKM Difference	Log_2_RPKM Fold change	FDR corrected p-value	Annotation - A. thaliana	Gene symbol – A. thaliana
Phenylpropanoid metabolism					
Nbv5tr6236806	67.0	2.7	<1.00E99	Phenylalanine ammonia-lyase-like	PAL2
Nbv5tr6372855	43.8	2.8	<1.00E99	Cinnamate-4-hydroxylase	C4H
Nbv5tr6215803	259.8	9.7	<1.00E99	4-coumarate-ligase-like	4CL
Nbv5tr6230272	50.5	3	<1.00E99	Cinnamoyl-CoA-reductase 1-like	CCR
Nbv5tr6354270	17.9	4.5	3.79E-04	Caffeic acid 5-hydroxyferulic acid o-methyltransferase	OMT
Nbv5tr6226062	888.8	3.4	<1.00E99	Caffeoyl-o-methyltransferase	CCoAOMT
Nbv5tr6216786	111.6	3.6	<1.00E99	Tyramine n-feruloyltransferase-like	AT2G39030
Nbv5tr6232617	1612.7	13.1	<1.00E99	Shikimate o-hydroxycinnamoyltransferase-like	AT1G32910
Nbv5tr6228735	662.3	10.5	<1.00E99	Laccase-12-like	LAC12
Nbv5tr6242242	970.1	10.4	<1.00E99	Laccase-3-like	LAC3
Nbv5tr6231653	27.4	8.7	<1.00E99	Laccase-4-like	IRX12
Nbv5tr6224494	774.6	co	<1.00E99	Peroxidase 27-like	AT3G01190
Nbv5tr6223363	56.4	co	<1.00E99	Peroxidase 3-like	PRX3
Nbv5tr6369451	23.1	co	<1.00E99	Peroxidase 72	AT5G66390
Nbv5tr6213304	263.4	11.2	<1.00E99	Peroxidase 64-like	AT5G42180
Nbv5tr6224466	269.8	12.8	<1.00E99	Casparian strip membrane protein 2-like	AT5G15290
Lipid Metabolism and transport					
Nbv5tr6200701	17.6	3.2	5.40E-13	Fatty acid amide hydrolase-like	FAAH
Nbv5tr6246533	40.4	4.6	<1.00E99	Acetyl- carboxylase 1-like	ACC2
Nbv5tr6237355	5.7	3.1	1.81E-04	Palmitoyl-acyl carrier protein chloroplastic-like	FATB
Nbv5tr6213323	176.4	2.6	<1.00E99	Very-long-chain-3-hydroxyacyl-dehydratase pasticcino 2	PAS2
Nbv5tr6201800	98.3	3.2	<1.00E99	3-ketoacyl- synthase 1	KCS1
Nbv5tr6211563	105.1	4.6	<1.00E99	3-ketoacyl- synthase 2-like	KCS2
Nbv5tr6222668	44.3	6	<1.00E99	3-ketoacyl- synthase 4-like	KCS4
Nbv5tr6220790	26.2	2	8.31E-15	3-ketoacyl- synthase 11-like	KCS11
Nbv5tr6384423	22.6	4.3	<1.00E99	Beta-ketoacyl reductase 1	KCR1
Nbv5tr6246568	39.8	5.5	<1.00E99	Long chain acyl- synthetase 4-like	LACS4
Nbv5tr6315453	58.0	7.6	<1.00E99	cytochrome family 86 subfamily A polypeptide 1	CYP86A1
Nbv5tr6237440	38.7	5.5	<1.00E99	cytochrome family 86 subfamily B polypeptide 1	CYP86B1
Nbv5tr6267165	28.6	9.5	<1.00E99	cytochrome family 86 subfamily A polypeptide 8	CYP86A8
Nbv5tr6240135	37.3	3.3	<1.00E99	Glycerol-3-phosphate acyltransferase 1-like	GPAT1
Nbv5tr6232718	34.3	8.9	<1.00E99	Glycerol-3-phosphate acyltransferase 5-like	GPAT5
Nbv5tr6218783	12.5	4.2	1.65E-05	Glycerol-3-phosphate 2-o-acyltransferase 6	GPAT6
Nbv5tr6201676	17.2	8.2	8.31E-15	Omega-hydroxypalmitate o-feruloyl transferase	AT1G03390
Nbv5tr6366084	16.2	6.2	2.97E-13	Omega-hydroxypalmitate o-feruloyl transferase	AT5G41040
Nbv5tr6234363	283.8	6.7	<1.00E99	Fatty acyl-CoA reductase 3-like	FAR3
Nbv5tr6222428	200.3	6.2	<1.00E99	GDSL esterase/lipase GELP51	AT2G23540
Nbv5tr6408655	27.6	6.6	<1.00E99	GDSL esterase lipase GELP96	AT5G37690
Nbv5tr6222744	28.8	4.1	<1.00E99	GDSL esterase lipase-like	AT1G71250
Nbv5tr6222730	391.0	9.5	<1.00E99	GDSL esterase lipase GELP38	AT1G74460
Nbv5tr6241864	72.6	2.3	<1.00E99	GDSL esterase lipase GELP72	AT3G48460
Nbv5tr6220564	28.7	7.7	<1.00E99	ABC transporter g family member 20	AT2G37360
Nbv5tr6368466	19.0	9.9	<1.00E99	ABC transporter g family member 1	AT2G39350
Nbv5tr6206538	29.5	4.7	<1.00E99	ABC transporter g family member 6	AT3G55090
Nbv5tr6391581	43.1	7.4	<1.00E99	ABC transporter g family member 23	AT5G19410
Nbv5tr6257293	165.6	11.8	<1.00E99	ABC transporter g family member 40	ABCG40
Nbv5tr6217020	21.9	7.9	<1.00E99	ABC transporter g family member 11	WBC11
Nbv5tr6222894	209.4	9.6	<1.00E99	Lipid transfer-like protein vas	AT5G13900
Nbv5tr6235917	81.1	7.1	<1.00E99	Non-specific lipid-transfer protein	AT1G05450
Cell wall metabolism					
Nbv5tr6207473	59.7	5.9	0.00E+00	Callose synthase 7-like	GSL07
Nbv5tr6258607	127.0	6.8	<1.00E99	Cellulose synthase-like protein d3	CSLD3
Nbv5tr6225388	94.2	2.9	6.28E-14	Expansin-like b1	EXLB1
Nbv5tr6236443	144.5	3.4	<1.00E99	Extensin-2	AT3G09925
Nbv5tr6235965	270.4	11	<1.00E99	Leucine-Rich Repeat extensin-like protein 6	AT3G22800
Nbv5tr6399534	116.8	7.5	<1.00E99	Glucuronoxylan 4-o-methyltransferase	AT1G33800
Nbv5tr6407350	25.3	7.3	<1.00E99	IRREGULAR XYLEM 15	AT5G67210
Nbv5tr6212953	90.4	6.4	<1.00E99	Mannan endo-beta-mannosidase 2-like	AT2G20680
Nbv5tr6256067	752.0	8.8	<1.00E99	Pectate lyase 8	AT3G07010
Nbv5tr6369798	2883.4	11.3	<1.00E99	Polygalacturonase	AT3G59850
Nbv5tr6390002	109.1	8.5	<1.00E99	polygalacturonase-like protein	AT3G61490
Nbv5tr6240865	26.6	4.6	<1.00E99	Probable arabinosyltransferase aradl	AT3G45400
Nbv5tr6205847	124.8	6.8	<1.00E99	Probable beta- -xylosyltransferase	GUT1
Nbv5tr6252885	79.6	6.2	<1.00E99	Probable beta- -xylosyltransferase	IRX14-L
Nbv5tr6249756	468.8	8.7	<1.00E99	Probable beta- -xylosyltransferase	IRX9
Nbv5tr6346223	1645.3	8.5	<1.00E99	Probable pectate lyase 15	AT4G13710
Nbv5tr6231824	59.7	7.9	<1.00E99	Probable pectinesterase 29	AT3G24130
Nbv5tr6217710	160.0	8.4	<1.00E99	Rhamnogalacturonate lyase b-like	AT1G09910
Nbv5tr6213987	86.3	6.4	<1.00E99	UDP-glucuronate:xylan alpha-glucuronosyltransferase-like	PGSIP1
Nbv5tr6223206	239.6	7.8	<1.00E99	UDP-glucuronate:xylan alpha-glucuronosyltransferase-like	PGSIP3
Transcription factors					
Nbv5tr6226272	12.3	6.2	4.31E-07	NAC domain containing protein 38	NACO38
Nbv5tr6292508	20.0	6.9	1.95E-11	NAC domain containing protein 58	NACO58
Nbv5tr6225931	8.5	3.3	1.45E-06	NAC domain containing protein 75	NAC075
Nbv5tr6335916	6.5	4.1	7.72E-05	NAC domain containing protein 80	NAC080
Nbv5tr6203800	12.9	4.3	1.55E-10	NAC domain containing protein 100	NAC100
Nbv5tr6229093	7.2	3	2.01E-05	transcription factor myb41-like	MYB41
Nbv5tr6214654	8.8	8.3	5.66E-08	transcription factor myb93-like	MYB93
Nbv5tr6223622	33.1	10.6	<1.00E99	transcription factor myb92-like	MYB92
Nbv5tr6342855	152.5	11.8	<1.00E99	transcription factor myb84-like	MYNA
Nbv5tr6218321	96.9	12.8	<1.00E99	transcription factor myb36-like	MYB36
Nbv5tr6237471	194.8	7	<1.00E99	transcription factor myb4-like	MYB4

Expression values were calculated using the Reads Per Kilobase Million (RPKM, Mortazavi et al. (2008)). RPKM difference reflects the absolute difference in expression (MYB68OE-EMPTY vector). Fold changes (FC) display the log_2_ RPKM ratio (MYB68 OE/Empty vector), with ∞ symbol displaying gene FC for which no expression was observed in leaves infiltrated with the empty vector. p-Values were adjusted using the Benjamini-Hochberg false discovery rate correction. Annotations were obtained as described in Legay et al. (2016). The full data RNA-Seq dataset is available in **Supplementary Table 4**.

Further, our gene ontology analysis highlighted a cluster of genes involved in the phenylpropanoid biosynthesis pathway. This included the phenylalanine ammonia-lysase (*PAL*), cinnamate-4-hydroxylase (*C4H*), 4-coumarate-ligase (*4CL*), hydroxycinnamoyl-Coenzyme A shikimate/quinate hydroxycinnamoyltransferase (*HCT*), caffeoyl-o-methyltransferase (*CCoAOMT*), the cinnamoyl-CoA reductase (*CCR*) and the caffeic acid 5-hydroxyferulic acid *O*-methyltransferase (*OMT*) (**Table 1**). According to the current knowledge, this gene expression pattern suggests that MdMYB68 is able to drive the synthesis of ferulic acid and guaiacyl lignin monomers (Fraser and Chapple, 2011; Woolfson et al., 2022). Furthermore, several laccase and peroxidase genes, which might affect the synthesis of the so-called polyphenolic/lignin-like suberin domain (Bernards, 2002; Graça, 2015; Serra and Geldner, 2022; Woolfson et al., 2022) were also induced by MdMYB68 (**Table 1**). As an example, the suberization-associated anionic peroxidase gene (*SAAP*), which displayed more than 63-fold increased expression, was previously described as crucial component of the suberization process (Bernards et al., 1999). Peroxidase 72, a cationic peroxidase involved in the lignin biosynthesis during the secondary cell wall biogenesis, has been also associated with apple russeting (Fernández-Pérez et al., 2015; Legay et al., 2015; André et al., 2022). Finally, we observed an increased expression of a tyramine N-feruloyltransferase, participating in the synthesis of feruloyl-tyramine, a suberin component which is proposed to contribute to pathogen tolerance in tomato (Kashyap et al., 2022), but the exact role of feruloyl-tyramines in the suberization process is still under investigation.

In addition to this observation, leaves expressing MdMYB68 displayed a massive increase of genes involved in the core lipid metabolic pathway. These included, on the one hand, several genes taking part in the synthesis of the long chain fatty acid (C16 and C18) and on the other hand, several acetyl-CoA carboxylase genes, which involve in the early step of the fatty acid synthesis. Number of genes coding acyl carrier protein thioesterase (FATB) were also induced. *FATB* genes, which are determinant for the synthesis of saturated fatty and their transport from the chloroplast to the cytosol, dispensed an increased expression in russeted apple skin, suggesting that they play a crucial role in the mobilization of fatty precursors for the synthesis of suberin building blocks (Bonaventure et al., 2003; Legay et al., 2015). MdMYB68 also triggered the expression of multiple 3-ketoacyl-CoA synthetase (*KCS1*, *KCS2*, *KCS4*, *KCS11*) and 3-ketoacyl-CoA reductase (*KCR1*) which belong to the fatty acid elongation complex and produce the very long chain fatty acid (VLCFA), a characteristic signature of the suberin monomers (Blacklock and Jaworski, 2006; Nomberg et al., 2022). KCS2/DAISY has been previously described as a major actor in the VLCFA biosynthesis in root suberin biosynthesis in Arabidopsis (Franke et al., 2009; Lee et al., 2009), whereas KCS4 displayed an enhanced expression in the suberized skin of the semi russeted ‘Cox Orange Pippin’ variety (Legay et al., 2017). Finally, a long chain Acyl-CoA synthetase 4 gene (*LACS4*), which is involved in the activation of the newly synthetized or elongated fatty acids was also induced in leaves expressing *MdMYB68*. To our knowledge, there is no identified suberin specific LACS in plants. In Arabidopsis, LACS4 has been associated with the synthesis of glycerolipids, wax or triacylglycerol and is highly expressed in the apple fruit pericarps, which altogether suggest that it might constitute a candidate being involved in the activation of dicarboxylic acids and/or ω-hydroxyacids (Zhao et al., 2021).

Omega-hydroxyacids constitute major components of suberin with α,ω-dicarboxylic acids and fatty alcohols. Their synthesis is mainly driven by *CYP86A1* and *CYP86B1* key genes, which can be considered as suberin synthesis markers (Höfer et al., 2008; Compagnon et al., 2009). In the present work, *CYP86A1* and *CYP86B1* genes displayed 194- and 44-fold increase in *N. benthamiana* leaves expressing *MdMYB68*, respectively. Interestingly, we also observed increase in expression of several *LACERATA*/*LCR*/*CYP86A8* genes. LCR involves in the ω-hydroxylation of C12 to C18:1 fatty acids and has been previously associated with the cutin synthesis in the epidermis (Wellesen et al., 2001). Beyond genes involved in the DCAs and ω-hydroxyacids biosynthesis, we also observed increased expression of several genes similar to the fatty acid reductase (*FAR3*), which are an important factors of the suberin specific fatty alcohols biosynthesis in plants (Domergue et al., 2010; Woolfson et al., 2018). Several isoforms of glycerol-3-phosphate acyltransferase (GPAT) displayed increased expression in leaves after MdMYB68 stimulation. This included the suberin specific *GPAT5* (482-fold increase) (Beisson et al., 2007) but also genes similar to GPAT1 (9.58-fold increase) and GPAT6 (18.6-fold increase). Interestingly, in tomato, GPAT6 has been previously associated with the synthesis of cutin oligomers precursors, but its clear role in the suberin biosynthesis has not been proposed, yet (Petit et al., 2016). Several BAHD-acyltansferase genes were upregulated after MdMYB68 overexpression. Among these, we found a gene similar to the Arabidopsis aliphatic suberin feruloyl transferase (ASFT/AT5G41040), which is responsible for the condensation of ferulic acid with fatty acids during the suberin monomer biosynthesis (Molina et al., 2009; Molina and Kosma, 2015; Nomberg et al., 2022). A large number of genes coding GDSL-motif esterase/lipase protein were also induced by MdMYB68. Among these we identified ortologous genes of the thale cress GELP38, GELP51, GELP72 and GELP96, which were highly induced in leaves expressing *MdMYB68*, with 700-, 73-, 4.8- and 95-fold increase, respectively. These were recently described as a crucial component of the final suberin assembly after the exportation of the suberin building blocks in the cell wall (Ursache et al., 2021). Finally, the putative transport of these suberin building blocks was also supported by the increased expression of several member of ATP-binding Cassette family G (ABCG) transporters, as well as genes coding for lipid transfer proteins (LTP). Among these ABCG2, ABCG6, ABCG20 were previously described as suberin specific transporters in Arabidopsis (Yadav et al., 2014).

Finally, *MdMYB68* transient expression additionally triggered higher expression of a large number of NAC- and MYB-family transcription factors. Genes similar to NAC038 and NAC058, which were previously co-expressed with suberin biosynthesis genes (Lashbrooke et al., 2015), displayed 73.34 and 119.54 fold increase in leaves expressing MdMYB68. Genes similar to the suberin regulators MYB92 and MYB93 were also massively induced with 1565.21- and 310.87-fold increases, whereas the ABA/drought-inducible MYB41 displayed only a 7.23-fold increase (Kosma et al., 2014; Legay et al., 2016; Shukla et al., 2021). A MYB36-like gene, which has been described as a major regulator of the casparian strip formation, displayed a massive increase in expression with a fold change equal to 7382.82 (Kamiya et al., 2015). Several copies of the well-known MYB4 phenylpropanoid biosynthesis regulator displayed an enhanced expression under MdMYB68 overexpression reaching 130.29-fold increase in expression (Jin et al., 2000; Andersen et al., 2021). Finally, a MYB84-like transcription factor, which have been recently associated with the root suberization, is also highly induced (Xu et al., 2022a).

### Changes in cell wall monosaccharide composition upon overexpression of *MdMYB68*

**Figure 3:**
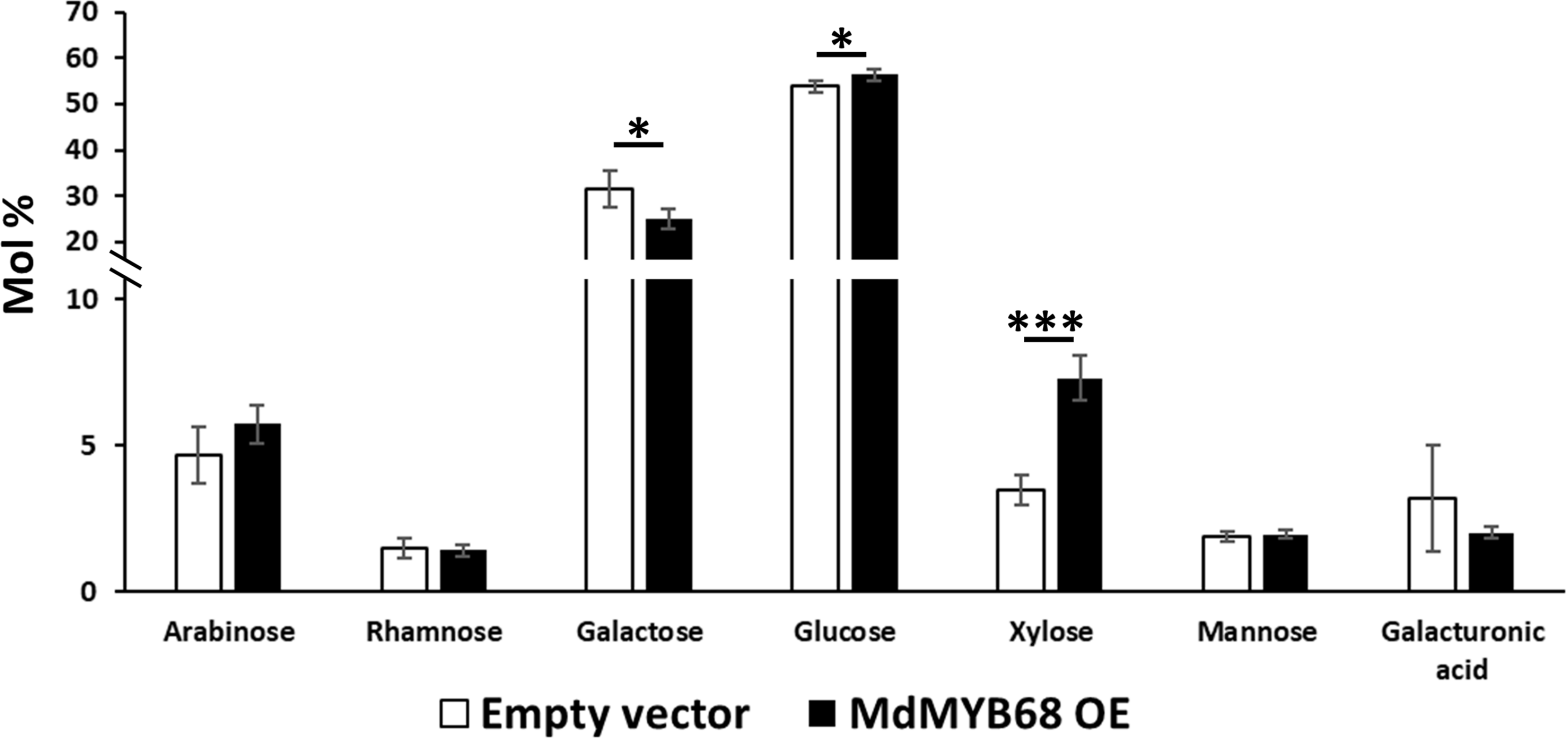
Monosaccharide composition of the total cell wall materials of control (white bars) and *MdMYB68*-overexpressing *N. benthamiana* leaves (black bars) collected 7 days after infiltration. Values are expressed as the mean ± standard deviation (SD) from four independent biological replicates. One and three asterisks indicate significant differences at *p* < 0.05 and *p* < 0.001, respectively.

As a first step towards the characterization of the tobacco cell walls, monosaccharide quantification was performed on total cell wall materials after one-step two-step hydrolysis. As expected by the adopted hydrolysis protocol, glucose (here deriving from both cellulose and matrix polysaccharides) was the most abundant monosaccharide (>50%). The pectinaceous nature of the tobacco leaves was confirmed by the higher abundance of galactose (representing >25% of the total sugars), with lower amounts of arabinose, xylose and galacturonic acid (between 3.2 and 7.3% of the total sugars; **Figure 3**). When comparing the leaves transformed with the empty vector (control) and those with the TF (*MdMYB68* OE): galactose decreased (from 31.51% ± 4.03% to 25.16% ± 2.21%), while xylose significantly increased upon overexpressing *MdMYB68* (from 3.46% ± 0.52% to 7.3% ± 0.76%) (**Figure 3**). Glucose increased slightly, but significantly, after overexpression of the TF (from 53.78% ± 1.25% to 56.39% ± 1.25%). These results suggest that the overexpression of the TF caused major modifications in the content of cell wall polysaccharides: these changes can be linked to hemicelluloses and, given its abundance in dicots’ primary walls, xyloglucan, (Scheller and Ulvskov, 2010) and to the pectic fraction.

**Figure 4:**
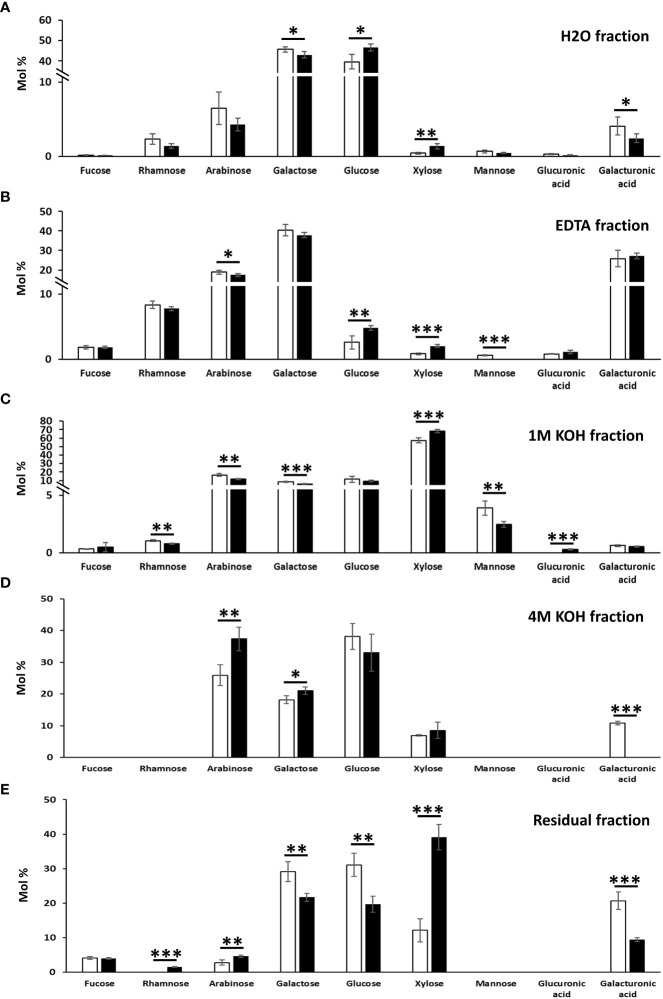
Monosaccharide composition of the cell wall fractions obtained by sequential extractions with hot water **(A)**, EDTA **(B)**, 1 M KOH **(C)**, 4 M KOH **(D)** and of the remaining residue **(E)** in *N. benthamiana* leaves agroinfiltrated with the empty vector and MdMYB68 constructs. Samples were collected 7 days after infiltration. Values are expressed as the mean ± SD from four biological replicates. One, two and three asterisks indicate significant differences at *p* < 0.05, *p* < 0.01, *p* < 0.001, respectively.

To confirm the classes of cell wall polysaccharides responsible for the changes observed in the content of monosaccharides, a sequential extraction of the cell wall components was then carried out (**Figure 4**). The hot water fraction of the control leaves, which typically extracts arabinogalactan proteins and loosely bound pectic polysaccharides, contained in majority glucose (>39%) and galactose (>40%), followed by arabinose (>4%), rhamnose (>1.4%) and arabinose (>4%) (**Figure 4A**).

The extraction of galactose, rhamnose and arabinose was indicative of rhamnogalacturonans (galacturonic acid, xylose for homogalacturonan and xylogalacturonan, respectively). In the *MdMYB68*-OE leaves, xylose abundance increased from 0.44% ± 0.18% in control samples to 1.37% ± 0.38% (**Figure 4A**). While no statistically significant differences were detected in the hot water fraction for rhamnose and arabinose (although both showed a tendency towards lower abundance in the transformed leaves), a significant decrease in galactose was evident after *MdMYB68*-overexpression. This result was in agreement with the analysis on the total cell wall materials (**Figure 3**) and confirmed alterations in the pectic fraction. To understand which regions of the pectic fraction were responsible for the changes observed, monosaccharide ratios were compared. The ratio galacturonic acid/rhamnose (indicating the proportion of “smooth” and “hairy” pectic regions) did not show significant changes (1.72 and 1.71 in control and TF-OE leaves), while the ratio galactose/rhamnose (providing an indication of rhamnogalacturonan I ramifications) denoted an increase after *MdMYB68* ectopic expression (from 19.3 in control samples to 30.4 in TF-OE leaves). The ratio arabinose/rhamnose, instead, did not reveal significant changes (2.7 and 3.0 in control and TF-OE leaves).

The EDTA fraction (**Figure 4B**) of the control leaves revealed the presence of the pectic monosaccharides galactose (>37.9%), galacturonic acid (>25.8%), arabinose (>17.4%), rhamnose (>7.8%), together with a lower abundance of fucose (>1.8%), xylose (>0.8%). Xylose and arabinose showed the same accumulation patterns as those observed in the hot water fraction: xylose significantly increased (from 0.81% ± 0.14% in control leaves to 2.02% ± 0.22% in *MdMYB68*-OE ones), while arabinose decreased (from 18.86% ± 0.90% to 17.42% ± 0.71%).

The 1M KOH fraction extracted typical hemicellulosic monosaccharides, with xylose being the highest in abundance (>57.4%), followed by arabinose (>11.9%), glucose (>9.4%), galactose (>5.8%), mannose (>2.5%) in the control leaves (**Figure 4C**). A statistically significant increase in xylose was detected, similarly to what already observed with the analysis on the total cell wall materials (**Figure 3**): the content increased from 57.4 ± 2.81% in control leaves to 68.4% ± 2.08% after *MdMYB68* overexpression (**Figure 4C**). Among other changes observed in the 1M KOH fraction, there was a significant decrease in mannose with 3.87% ± 0.63% in the control to 2.46% ± 0.26% after *MdMYB68* overexpression.

In the 4M KOH fraction obtained from leaves expressing MdMYB68, arabinose was the most abundant (37.36%) followed by glucose (30,06%), galactose (21.02%) and xylose (8.56%) (**Figure 4D**). Significant differences were observed only for arabinose (25.9% ± 3.27% in the control to 37.36% to ± 3.75%), galactose (18.16% ± 1.25% in the control to 21.02% to ± 1.21%) and galacturonic acid (10.78% ± 0.58% in the control to nothing).

Finally, the alkali-insoluble cell wall residue of the control leaves contained in majority glucose (>19.7%), xylose (>12%) and galacturonic acid (>9.4%), followed by minor amounts of fucose (>3.9%) and arabinose (>2.8%) (**Figure 4E**). A massive increase in xylose was observed after *MdMYB68* overexpression (12.14% ± 3.72% in the control leaves to 39.12% ± 3.34%). Other significant differences were observed for glucose (31.11% ± 3.36% in the control leaves to 19.75% ± 2.32%), galactose (29.14% ± 2.84% in the control leaves to 21.68% ± 1.22%), galacturonic acid (20.69% ± 2.51% in the control leaves to 9.41% ± 0.57%), arabinose (2.78% ± 0.72% in the control leaves to 4.6% ± 0.32%) and finally rhamnose (not detectable in the control leaves to 1.49% ± 0.15%).

### MdMYB68 triggered the suberin precursor accumulation and suberin deposition

**Figure 5:**
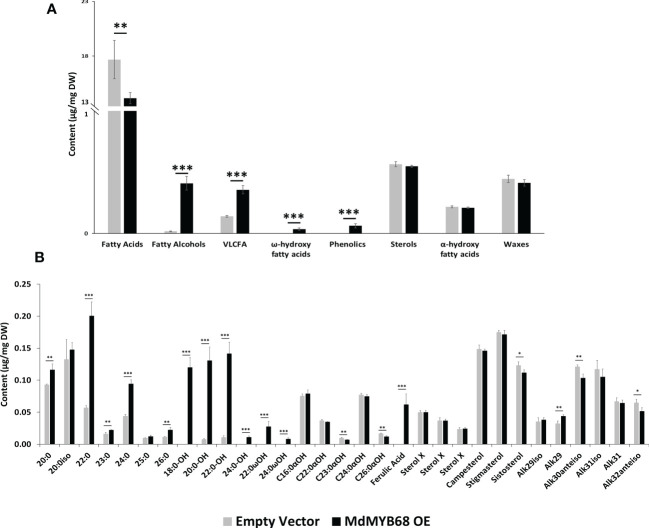
GC-MS analysis performed from the soluble lipid fraction obtained from *Nicotiana benthamiana* leaves collected 7 days after agroinfiltration with the MdMYB68 construct (MdMYB68 OE) or the Control/empty vector construct (Empty vector). 4 biological replicates were used (n=4). **(A)** content gathered by lipid subclasses, **(B)** fatty acids, fatty alcohols, ω- and α-hydroxy fatty acids, ferulic acid, sterols, and waxes. C16 to C18 fatty acid contents are displayed in the **Supplementary Figure 3**. Values are expressed as the mean ± SD from four biological replicates. One, two and three asterisks indicate significant differences at *p* < 0.05, *p* < 0.01, *p* < 0.001, respectively.

**Figure 6:**
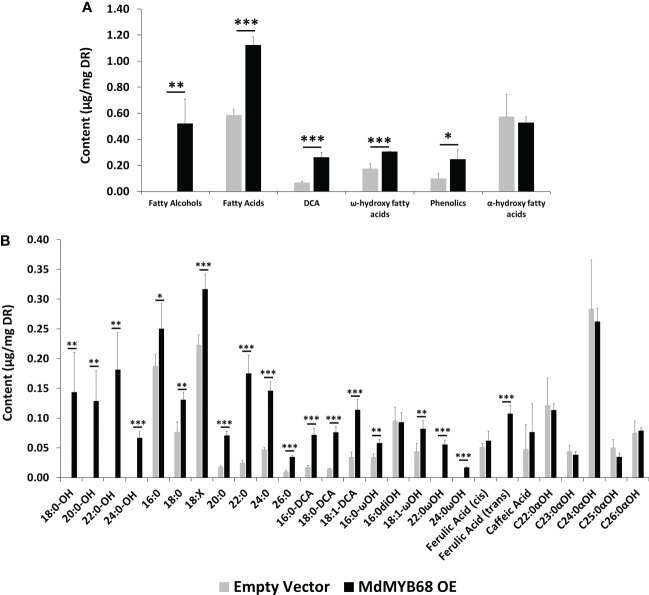
GC-MS analysis performed from the trans-esterified lipid fraction obtained from *Nicotiana benthamiana* leaves samples collected 7 days after agroinfiltration with the MdMYB68 construct (MdMYB68 OE) or the Control/empty vector construct (Empty vector). 4 biological replicates were used (n=4). **(A)** content gathered by lipid subclasses, **(B)** fatty acids, fatty alcohols, ω- and α-hydroxy fatty acids, ferulic acid, sterols, and waxes. Values are expressed as the mean ± SD from four biological replicates. One, two and three asterisks indicate significant differences at *p* < 0.05, *p* < 0.01, *p* < 0.001, respectively.

A fluorol yellow 088 staining was first performed to visualize the formation of suberin lamellae in *N. benthamiana* leaves 7 days after infiltration with the MdMYB68 construct (**Supplementary Figure 2**). Adaxial side of the leaves displayed higher fluorescence localized in cell wall of palisade cells in leaves expression MdMYB68 whereas only a week signal was observed in the control leaves (**Supplementary Figures 2C, D**). This was further supported by observation made in parenchymal cells (**Supplementary Figures 2E, F**) where higher fluorescence was also observed upon MdMYB68 expression. To further validate observations made in our gene expression profiling study, a GC-MS analysis of lipids was performed on both soluble and polymer fractions collected after 7 days from leaves infiltrated with the MdMYB68 construct and the empty vector. First, increased contents in trans ferulic acid (p-value<0.001), were observed in *N. benthamiana* leaves expressing *MdMYB68* whereas these were not detectable in control leaves (**Figures 5**, **6**). This observation is in accordance with the gene expression data and support the fact that MdMYB68 can regulate the phenylpropanoid pathway.

The analysis of the lipid compound family accumulated in the solvent soluble fraction showed that fatty alcohols, very long chain fatty acids, and ω-hydroxyacids contents increased in leaves expressing *MdMYB68* whereas sterols, α-hydroxy fatty acids (typically found in sphingolipids), and waxes remained unchanged (**Figure 5A**). It is particularly noteworthy that these increased contents were mainly caused by an accumulation of compounds with very long chain aliphatic backbones. The C20, C22, C24 and C26 fatty acid contents were respectively increased 1.25-, 3.52-, 2.16- and 1.97-fold in leaves expressing *MdMYB68* compared to the control leaves (**Figure 5B**). Inversely, C16, C16:3 and C18:x fatty acid contents decreased in response to *MdMYB68* overexpression, suggesting a possible remobilization of the core fatty acid pool (**Supplementary Figure 3**). In leaves expressing *MdMYB68*, long and very long chain fatty alcohols, which are synthesize by Fatty Acyl Reductases (FARs), displayed increased contents with a specific accumulation of C18, C20, C22, and more marginally C24 aliphatic backbones. Altogether, these data suggested, as hypothesized from the RNA-seq data, that MdMYB68 was able to trigger the synthesis of the very long chain aliphatic precursors and downstream suberin specific compounds and their associated waxes.

The analysis of the polymerized lipid fraction revealed a significant increase in total fatty alcohols, fatty acids, dicarboxylic acids and ω-hydroxyacids in *N. benthamiana* leaves expressing MdMYB68 whereas the α-hydroxy fatty acid (typically found in sphingolipids) content did not changed (**Figure 6A**). Interestingly, fatty alcohols, with aliphatic chain size ranging from C18 to C24 were only observed in leaves expressing *MdMYB68* (**Figure 6B**). C20, C22, C24 and C26 fatty acids displayed the highest fold changes, with FC=3.94, 7.16, 3.12 and 3.51, respectively. A similar increase was observed for ω-hydroxy fatty acid contents for which an accumulation of C16, C18:1, C22 and C24 was observed. Interestingly C22 and C24 ω-hydroxyacids were only detectable in response to MdMYB68 expression. Finally, C16, C18 and C18:1 dicarboxylic acid accumulated upon MdMYB68 expression with 4.13, 5.20 and 3.28 fold increases, respectively. All the fatty acid derivatives which content increased significantly are well-known as crucial components of the suberin polymer backbone (Serra and Geldner, 2022; Woolfson et al., 2022). In contrast, dihydroxypalmitate (16:0diOH), a typical cutin monomer, remained unaffected in response to *MdMYB68* expression (**Figure 6B**).

### Histological study revealed modifications in the cell wall of leaves infiltrated with MdMYB68

**Figure 7:**
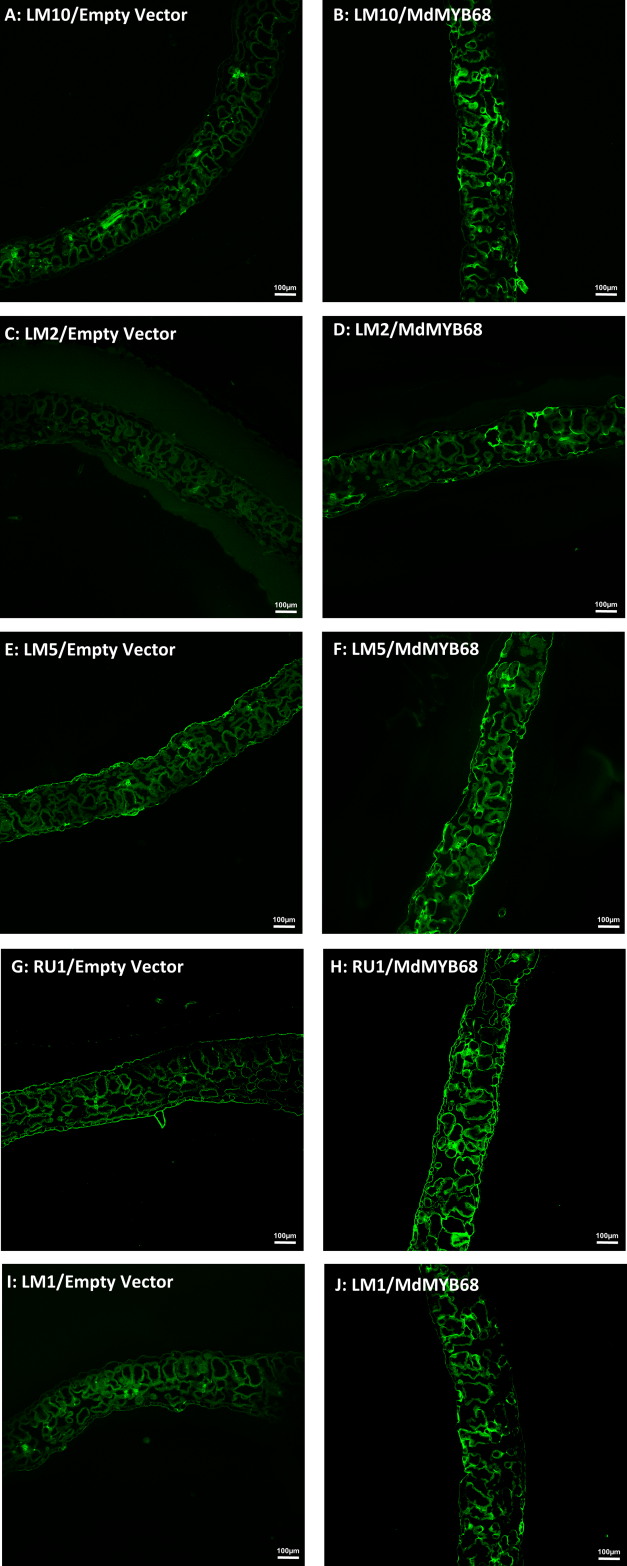
Immunodetection performed from *N. benthamiana* leaf samples collected 7 days after agroinfiltration with the Empty vector (left side) and MdMYB68 (right side) construct using the following antibodies: LM10 specific for unsubstituted β-1,4-Xylan **(A, B)**, LM2 specific for carbohydrates containing β-linked glucuronic **(C, D)**, LM5 specific for β-1,4-D-galactan **(E, F)**, RU1 specific for unbranched regions of rhamnogalacturonan-I **(G, H)**, LM1 specific for extensins/HRGP **(I, J)**.

To further support our hypothesis built from the RNA-seq data and cell wall composition analysis, we performed a histological analysis using confocal microscopy associated with monoclonal antibodies targeting different cell wall components on samples collected 7 days after agroinfiltration with the empty vector and MdMYB68 constructs. In control leaves infiltrated with the empty vector construct, the LM10, which is specific to unsubstituted β-1,4-xylan (McCartney et al., 2005), only showed fluorescence in vessels, whereas in the leaves infiltrated with the MdMYB68 construct a higher intensity was observed in the entirety of cell walls of parenchymal cells. This supports the fact that a strong modification/synthesis of xylans was triggered by MdMYB68 (**Figures 7A, B**; **Supplementary Figure 4**). Staining with the LM2 antibody, recognizing epitopes of carbohydrates containing β-linked glucuronic acid, also revealed a higher, but patchier fluorescence, in parenchymal cells expressing *MdMYB68* (**Figures 7C, D**). This suggest that an accumulation of arabinogalactan protein, important players in the cell wall-membrane continuum and interactions with pectins, occurred. Similarly, the LM5 antibody staining, targeting pectic polysaccharides (β-1,4-galactan), suggests that a strong modification/accumulation of pectins occurred in response to the ectopic expression of *MdMYB68* (**Figures 7E, F**; **Supplementary Figure 4**). Using the INRA-RU1 antibody, we observed a massive increase in fluorescence in leaf samples expressing *MdMYB68* (**Figures 7G, H**; **Supplementary Figure 4**). RU1 is specifically recognizing unbranched regions of rhamnogalacturonan-I and requires at least 6 disaccharide backbones (optimally 7) repeats for binding. This suggests that MdMYB68 triggered the increase in rhamnogalacturonan-I long branching which might strongly affect the cellulose/pectin network interaction. Finally, with respect to the large number of upregulated extensins genes observed in the gene expression data, leaf samples were stained using the LM1 antibody, which recognizes extensins/hydroxyproline rich glycoprotein (HRGP; Mishler-Elmore et al., 2021). Again a stronger fluorescence was observed in leaves expressing *MdMYB68* suggesting that a massive cell wall remodeling and/or expansion occurred (**Figures 7I, J**; **Supplementary Figure 4**).

## Discussion

The transient expression of *MdMYB68* in *N. benthamiana* leaves triggered a massive alteration of multiple pathways including the cell wall matrix polymers, phenylpropanoid and lipid metabolisms. First, major changes in expression were observed for genes involved in both the phenylpropanoid and lipid metabolisms, and those effects were concordant with strong alterations in the lipid contents of leaves infiltrated with the MdMYB68 construct. A significant number of phenylpropanoid biosynthesis and peroxidase genes were induced during the MdMYB68 overexpression (**Table 1**; **Supplementary Table 4**), suggesting that this transcription factor can regulate the synthesis of the aromatic components of suberin. This observation was confirmed by the GC-MS analysis, which highlighted significant and massive increase of trans ferulic acid in the soluble and polymerized fractions, respectively. This suggests that MdMYB68 regulates the synthesis ferulic acid and derivatives, which are either integrated in the suberin backbone or present as suberin associated waxes. RNA-Seq data confirmed this observation as gene similar to the feruloyl-CoA acyltransferase and tyramine n-feruloyltransferase were induced by MdMYB68. In Arabidopsis, the feruloyl-CoA acyltransferase is involved in the synthesis of suberin oligomers through condensation of DCAs and ω-hydroxyacids with ferulic acid (Molina et al., 2009). The tyramine n-feruloyltransferases are responsible for the synthesis of feruloyl-tyramines, which have been identified as an aliphatic suberin component in plants and associated with abiotic stress tolerance (Graça, 2015; Kashyap et al., 2022).

Transcriptomic data revealed an extremely clear suberin biosynthesis signature with an increased expression of key genes such as the KCS, involved in the elongation of long chain fatty acid to very long chain fatty acid (VLCFA, >C18). Our GC-MS results confirmed this observation as multiple compounds family displayed specific increase of their C20, C22, C24 and sometimes C26 members (**Figures 5**, **6**). This was observed for fatty acids and fatty alcohols in soluble fraction as well as for fatty alcohols, fatty acids and ω-hydroxyacids in the depolymerized fraction. VLCFA are commonly found in plants and have a large diversity of function in (i) membrane lipids (sphingolipids, phospholipids), (ii) storage lipids as triacylglycerols or (iii) surface lipids including waxes and suberin monomers such ω-hydroxyacids and α,ω-dicarboxylic acids (Batsale et al., 2021).

Our data also showed increased expression of *CYP86A1* and *CYP86B1*, which are responsible for the fatty acid ω-hydroxylation leading to suberin specific ω-hydroxyacids (Höfer et al., 2008; Compagnon et al., 2009). This was confirmed by our GC-MS analysis results, which showed accumulation of members of these compound family in the suberin polymer. It is noteworthy that several copies of gene similar to LCR/CYP86A8 were also upregulated. Previous studies showed that LCR is mainly involved in the cutin biosynthesis (Wellesen et al., 2001), but our data suggest that it might also be involved in the fatty acid ω-hydroxylation during the suberization process. The increased expression of the well-known suberin specific GPAT5, which is involved in the formation of acyl-glycerol oligomers, also strongly suggests that MdMYB68 regulates suberin biosynthesis (Beisson et al., 2007). However, GPAT6, which was previously associated with cutin biosynthesis, was also induced by the MdMYB68 overexpression (Petit et al., 2016). Increased GPAT6 expression was also observed in *N. benthamiana* leaves expressing the suberin biosynthesis regulator MdMYB93 (Legay et al., 2016). Further investigations could be conducted to understand whether in addition to the GPAT5, the GPAT6 also plays a role in the suberin monomer oligomerization.

Beside ω-hydroxyacids and DCAs, primary alcohols constitute among the most important components of the suberin polymer and associated waxes i.e. alkyl-hydroxycinnamate. (Delude et al., 2016; Domergue and Kosma, 2017). Looking at the gene expression data, several genes coding for fatty acyl-CoA reductase (FAR) were induced by MdMYB68 with a concomitant massive accumulation of fatty alcohols in the soluble fraction as well as a *de novo* accumulation of fatty alcohol was observed in the polymer fraction. These fatty alcohols displayed aliphatic chains ranging from C18 to C24 being defined multiple times as a common suberin signature suggesting that MdMYB68 tightly regulates their synthesis (Domergue et al., 2010; Delude et al., 2016).

Apart from genes responsible for the synthesis of suberin aromatic and aliphatic monomers, transcriptomic data highlighted number of genes involved in transport, particularly ATP-binding cassette family G (ABCG) transporters which have previously been associated with suberin deposition in plants. In Arabidopsis, ABCG6 and ABCG20 were described as crucial actors of the suberin deposition in seedcoat and root endodermis whereas ABCG23 is co-expressed with suberin biosynthesis genes (Yadav et al., 2014). In potato ABCG1 has been described as a crucial component of the suberin deposition in the potato tuber periderm. Indeed, *ABCG1-*RNAi lines displayed strong decrease of ω-hydroxyacid-C18:1, DCA-C18:1 as well as fatty acid and fatty alcohols with aliphatic chain from C24 to C28 (Landgraf et al., 2014). ABCG11 has been shown to be involved in both cutin and suberin deposition in Arabidopsis (Panikashvili et al., 2010). As previously mentioned, MdMYB68 seemed to share a similar role with MdMYB93 as ABCG1, ABCG11, ABCG23 genes were induced by these two transcription factors in *N. benthamiana* leaves (Legay et al., 2016, this study).

Finally, a recent study described for the first time the involvement of the GDSL-type esterase/lipase proteins (GELP) in the suberin assembly and degradation (Ursache et al., 2021). As observed for MdMYB93 or in russeted apple skin (Legay et al., 2015; Legay et al., 2016), MdMYB68 expression triggered the downstream expression of a large number of GELPs in *N. benthamiana*. Among these, we were able to identify orthologous GELP genes in Arabidopsis, namely the GELP38, GELP51, GELP72 and GELP96. According to Ursache et al. (2021), these upregulated GELPs are associated with the suberin polymerization in root endodermis, which suggests that MdMYB68 is also able to regulate the suberin final assembly.

To further strengthened the crucial role of MdMYB68 in the regulation of the suberin biosynthesis, we investigated its downstream regulatory cascade. The NAC038 and NAC058 transcription factors, despite not fully described, were shown to be co-expressed with suberin biosynthesis genes in apple fruit skin and with a number of suberin biosynthesis regulator (Legay et al., 2015; Lashbrooke et al., 2016; Legay et al., 2016). This suggests one the one hand that these two transcription factors might be part of suberin regulatory network, and on the other hand that MdMYB68 acts in a similar way compared to previously described suberin regulators. This observation is further confirmed by the induction of MYB-domain transcription namely MYB93, MYB92, or MYB41, which were previously described as suberin master regulator in Arabidopsis and apple (Kosma et al., 2014; Legay et al., 2016; To et al., 2020; Shukla et al., 2021). This suggests that MdMYB68 might be an upstream regulator compared to these latter transcription factors, which is in accordance with a recent study, which proposed a modeling of the suberin deposition regulatory network in Arabidopsis (Xu et al., 2022a). Indeed, in this work, authors suggest that AtMYB68 is a top tier regulator of the suberin deposition process and thus able to induce the expression of MYB9, MYB93, MYB92, MYB41 and also MYB52, which has been recently described as a crucial regulatory component of the suberin aromatic domain biosynthesis. However, our transcriptomics work also showed that MdMYB68 triggered the expression of MYB36 and MYB84, which is not in accordance with statements made in the same article and suggests that other components of the suberin regulatory network might interact in this complex scheme in apple (Kamiya et al., 2015; Xu et al., 2022a).

Apart from the suberin specific alteration of lipid and phenylpropanoid metabolism triggered by MdMYB68, we investigated for the first-time the impact of gene expression changes on cell wall components upon suberization. Indeed, the present results showed that the cell wall composition was strongly affected by MdMYB68 overexpression. Major alterations of galactose, glucose and xylose and galacturonic acid were observed in the global cell wall analysis (**Figure 4**). The high abundance of glucose could be indicative of starch; however, the extraction protocol degraded selectively starch by the action of amylases, therefore any contribution by this polymer is negligible. A possible source of glucose is the hemicellulose glucomannan: some mannose was indeed present in this fraction and this class of polymers was previously extracted with hot water in coffee leaves (Lima et al., 2013). Glucose could also derive from callose: in support of this hypothesis is the increased expression of a callose synthase 7 gene (contig Nbv5tr6207473) after overexpressing *MdMYB68* in tobacco leaves (**Table 1**, **Supplementary Table 4**). Notably, this gene is responsible for the deposition of callose in the plasmodesmata of sieve plates and ultimately determines the correct development of the phloem tissue (Xie et al., 2011). The dynamics of callose deposition at plasmodesmata may be important in tobacco leaves overexpressing *MdMYB68*: severing of plasmodesmata is known to occur as suberin lamellae are deposited (Ma and Peterson, 2000) and callose may play a role in symplastic isolation during ectopic suberin deposition. In addition, callose was shown to play the function of semipermeable apoplastic barrier in muskmelon endosperm envelope in a manner analogous to suberin or lignin (Yim and Bradford, 1998). In the context of the present investigation, callose deposition may be a consequence of the ectopic expression of a suberin biosynthesis master-regulator in a tissue primarily composed of parenchyma cells possessing a primary cell wall. The deposition of this glucose polymer could thus contribute to cell wall-related changes accompanying suberin deposition. We might also hypothesize that MdMYB68 plays a role in the sealing of cells damaged by microcracks occurring in russeted apples, reducing plasmodesmata trafficking concomitantly with the suberin deposition (Knoche et al., 2011; Khanal et al., 2012). Finally, the cell wall modification process was also supported by the increased expression of a large number of extensins (**Table 1**, **Supplementary Table 4**), further confirmed by the immunostaining (**Figure 7**, **Supplementary Figure 4**). Little is known about their role in the suberin deposition, but extensins might be involved in the complex process of cell wall remodeling particularly through enhanced crosslinking with other cell wall components including pectins, lignin or arabinogalactan proteins, which were also affected by MdMYB68 (**Figure 7**, Mishler-Elmore et al., 2021).

The decrease in galacturonic acid and increase in xylose observed in the hot water fraction (**Figure 4A**) possibly reflect modification of the homogalacturonan and xylogalacturonan contents, respectively. Indeed, three contigs (Nbv5tr6360507, Nbv5tr6404041 and Nbv5tr6240865) annotated as orthologs of thale cress *At3g45400* showed an increased expression after agroinfiltration with the *MdMYB68* construct (**Table 1**, **Supplementary Table 4**). *At3g45400* codes for an exostosin family protein and BLASTp analysis revealed some sequence homology (E-value 7E-17) to the functionally validated xylogalacturonan xylosyltransferase *XGD1*-*At5g33290* (Jensen et al., 2008). The role of xylogalacturonan still needs to be fully unveiled: the presence of xylose substitution on homogalacturonan chains prevents the formation of the Ca^2+^-dependent “egg-box” structure thereby favoring loosening, but one must also consider that xylogalacturonan is resistant to the action of endo-polygalacturonases (Jensen et al., 2008).

Genes involved in pectin modifications (pectin lyases and esterases), which were showed to be up-regulated in tobacco leaves overexpressing the suberin regulator *MdMYB93* (Legay et al., 2016), were also induced by MdMYB68. As a result, the contigs Nbv5tr6369798, Nbv5tr6292424, Nbv5tr6369799 and Nbv5tr6315850 coding for polygalacturonases showed an increased expression >900-fold upon overexpression of *MdMYB68* (**Table 1**, **Supplementary Table 4**). A remodeling of the pectic network relying on one hand on loosening (by upregulation of pectin hydrolases and promotion of homogalacturonan substitution with xylose) and, on the other hand, on increased resistance to hydrolysis by xylose-rich homogalacturonan subregions thus seems to accompany the suberization process. This was supported by the increased galactose/rhamnose ratio which suggests that rhamnogalacturonan I had more ramifications upon the ectopic expression of *MdMYB68*. This can result in stronger interactions of pectin with cellulose and xyloglucan (Zykwinska et al., 2005) and in the ultimate strengthening of the primary cell wall (Le Mauff et al., 2017).

The increase in xylose, observed in the 1M KOH fraction could be linked to xyloglucan, the major hemicellulose in primary walls. By looking at the RNA-Seq dataset, several genes involved in xylan biosynthesis (orthologs of *IRX9*, *IRX14L* and *GUT1*; Brown et al., 2009; Wu et al., 2010) were upregulated in the *MdMYB68*-OE leaves (**Table 1**, **Supplementary Table 4**). The significant increase in glucuronic acid in the transformed leaves is a clear confirmation of the hypothesis that xylan deposition is promoted by the expression of *MdMYB68*. Additionally, contigs annotated as orthologs of thale cress *PGSIP1*-*GUX1* and *PGSIP3*-*GUX2*, which were previously shown to catalyze glucuronic acid addition to xylan in thale cress (Rennie et al., 2012), increased after overexpression of *MdMYB68* (**Table 1**, **Supplementary Table 4**). Taken together, these results suggest an enhanced xylan biosynthesis, as evidenced by the parallel increase in the xylose backbone and glucuronic acid residues of the side chains. A decrease in mannose was also observed in the 1M KOH fraction. This monosaccharide likely derives from glucomannan whose mannose residues were shown to mediate the formation of lignin-carbohydrate complexes *via* an α-ether bond (Nishimura et al., 2018). The specific decrease of mannose upon overexpression of the *MdMYB68* may be mediated by mannanases: in the RNA-Seq dataset, two ortologs of thale cress mannanase were also upregulated after agroinfiltration of *MdMYB68*, i.e., *At2g20680* and *At5g66460* (**Table 1**, **Supplementary Table 4**). The decrease in mannose residues may trigger modifications in the lignin-hemicellulose network during ectopic suberin deposition.

Interestingly, the 4M KOH fraction extracted more arabinose compared to 1M KOH (**Figures 4C,D**). This may be due to the association of arabinose to the amorphous regions of cellulose (Li et al., 2013). The abundance of arabinose branching was correlated with a lower cellulose crystallinity and a higher digestibility of *Miscanthus* lignocellulose (Li et al., 2013). A significant higher abundance of arabinose was observed in the transformed leaves (**Figure 4D**), together with a higher arabinose/xylose ratio. The arabinose/xylose ratio is indicative of xylan branching: higher values correspond to a polymer with a higher degree of branching. The overexpression of *MdMYB68* may therefore cause modifications in the abundance of xylan, as well as in its branching. It remains to be verified whether these changes in xylan structure have any implications on the degree of cellulose crystallinity.

The remaining alkali-insoluble cell wall residue displayed a large majority of glucose, xylose and galacturonic acid (**Figure 5E**). These monosaccharides are indicative of cellulose and remaining hemicelluloses (xyloglucan, xylan). The significant increase in xylose observed in the other fractions was confirmed here too, together with the decrease in galactose. We nevertheless suspect that part of the hemicellulose components, particularly xyloglucans and xylans, might be less extractable by the 1M and 4M KOH sequential extractions steps due to the ectopic deposition of suberin in the cell wall. Further work should be performed to validate this hypothesis. A significant decrease in glucose was also observed in the alkali-insoluble residue. Interestingly, members of the cellulose synthase gene-like family *CSLD3* showed, however, strong induction after *MdMYB68* overexpression (**Table 1**, **Supplementary Table 4**). *CSLD3* was shown to be involved in the synthesis of (1→4)-β-glucan polysaccharides, its activity could be replaced by *CesA6* (Park et al., 2011) and a CEAS6 chimera containing the catalytic domain of CSLD3 could rescue a *cesa6* mutant (Yang et al., 2020).

Although, it would have been ideal to overexpress MdMYB68 in apple leaves or fruits to determine its *in vivo* function in apple, heterologous transient expression in *N. benthamiana* constituted again a powerful and efficient tool for the validation of suberin biosynthesis related transcription factors in apple. Altogether, this study suggests that MdMYB68 plays a major role in the regulation of the suberin deposition in russeted apple skin. In addition to activate the lipid and phenylpropanoid pathways associated with the biosynthesis of the suberin aromatic and aliphatic components, our present transcriptomic data revealed that MdMYB68 affected the expression of many cell wall related genes. This suggests that MdMYB68 might be able to regulate a cell wall remodeling process, which might represent a crucial step preceding suberin deposition in apple, and most probably in plants (Legay et al., 2015; Legay et al., 2016; André et al., 2022; Serra and Geldner, 2022). This role is further sustained by the changes of the different cell wall components we observed during the suberization process. Our cell wall analytical pipieline specifically suggests that MdMYB68 might be able to alter pectins and hemicellulose, and in particular the xylans composition. Here, we presented one more crucial suberin regulator. MdMYB68, which triggered the activation of pathways already observed in our previous gene expression studies, namely the phenylpropanoids and lipid pathways, as well as cell wall modifying pathways. Further studies should aim at deciphering the top/bottom or upstream/downstream regulatory cascade regulating timely these different pathways that collectively lead to the formation of suberin lamellae at the inner side of the well wall.

## Data availability statement

The datasets presented in this study can be found in online repositories. The names of the repository/repositories and accession number(s) can be found below: https://www.ncbi.nlm.nih.gov/geo/query/acc.cgi?acc=GSE220694, GSE220694.

## Author contributions

XX involved in the genomic work, cell wall analysis, data analysis, microscopy, paper writing and refinement. GG involved in the project ideation, genomic work, cell wall analysis, data analysis, paper writing and refinement. FD involved in the lipids analysis, data analysis, paper writing and refinement. OB-G involved in the genomic work, data analysis, paper writing and refinement. EC involved in the lipids analysis and data analysis. RB involved in the microscopy, data analysis, paper writing and refinement. KS involved in the cell wall analysis, data analysis, paper writing and refinement. J-FH involved in the project ideation, paper writing and refinement. SL involved in the project ideation, genomic work, cell wall analysis, data analysis, paper writing and refinement. All authors contributed to the article and approved the submitted version.

## References

[B1] Andersen T. G. Molina D. Kilian J. Franke R. B. Ragni L. Geldner N. (2021). Tissue-autonomous phenylpropanoid production is essential for establishment of root barriers. Curr. Biol. 31, 965–977.e5. doi: 10.1016/j.cub.2020.11.070 33529644

[B2] André C. M. Guerriero G. Lateur M. Charton S. Leclercq C. C. Renaut J.. (2022). Identification of novel candidate genes involved in apple cuticle integrity and russeting-associated triterpene synthesis using metabolomic, proteomic, and transcriptomic data. Plants 11, 289. doi: 10.3390/plants11030289 35161271 PMC8838389

[B3] Baggerly K. A. Deng L. Morris J. S. Aldaz C. M. (2003). Differential expression in SAGE: accounting for normal between-library variation. Bioinforma. Oxf. Engl. 19, 1477–1483. doi: 10.1093/bioinformatics/btg173 12912827

[B4] Batsale M. Bahammou D. Fouillen L. Mongrand S. Joubès J. Domergue F. (2021). Biosynthesis and functions of very-Long-Chain fatty acids in the responses of plants to abiotic and biotic stresses. Cells 10, 1284. doi: 10.3390/cells10061284 34064239 PMC8224384

[B5] Behr M. Legay S. Žižková E. Motyka V. Dobrev P. I. Hausman J.-F.. (2016)Studying secondary growth and bast fiber development: The hemp hypocotyl peeks behind the wall (Accessed May 17, 2022). 10.3389/fpls.2016.01733 PMC5114303 27917184

[B6] Beisson F. Li Y. Bonaventure G. Pollard M. Ohlrogge J. B. (2007). The acyltransferase GPAT5 is required for the synthesis of suberin in seed coat and root of arabidopsis. Plant Cell 19, 351–368. doi: 10.1105/tpc.106.048033 17259262 PMC1820950

[B7] Bernards M. A. (2002). Demystifying suberin. Can. J. Bot. 80, 227–240. doi: 10.1139/b02-017

[B8] Bernards M. A. Fleming W. D. Llewellyn D. B. Priefer R. Yang X. Sabatino A.. (1999). Biochemical characterization of the suberization-associated anionic peroxidase of potato. Plant Physiol. 121, 135–146. doi: 10.1104/pp.121.1.135 10482668 PMC59361

[B9] Bindea G. Mlecnik B. Hackl H. Charoentong P. Tosolini M. Kirilovsky A.. (2009). ClueGO: A Cytoscape plug-in to decipher functionally grouped gene ontology and pathway annotation networks. Bioinformatics 25, 1091–1093. doi: 10.1093/bioinformatics/btp101 19237447 PMC2666812

[B10] Blacklock B. J. Jaworski J. G. (2006). Substrate specificity of arabidopsis 3-ketoacyl-CoA synthases. Biochem. Biophys. Res. Commun. 346, 583–590. doi: 10.1016/j.bbrc.2006.05.162 16765910

[B11] Bonaventure G. Salas J. J. Pollard M. R. Ohlrogge J. B. (2003). Disruption of the FATB gene in arabidopsis demonstrates an essential role of saturated fatty acids in plant growth. Plant Cell 15, 1020–1033. doi: 10.1105/tpc.008946 12671095 PMC152346

[B12] Brown D. M. Zhang Z. Stephens E. Dupree P. Turner S. R. (2009). Characterization of IRX10 and IRX10-like reveals an essential role in glucuronoxylan biosynthesis in arabidopsis. Plant J. 57, 732–746. doi: 10.1111/j.1365-313X.2008.03729.x 18980662

[B13] Cohen H. Fedyuk V. Wang C. Wu S. Aharoni A. (2020). SUBERMAN regulates developmental suberization of the arabidopsis root endodermis. Plant J. Cell Mol. Biol. 102, 431–447. doi: 10.1111/tpj.14711 32027440

[B14] Compagnon V. Diehl P. Benveniste I. Meyer D. Schaller H. Schreiber L.. (2009). CYP86B1 is required for very long chain omega-hydroxyacid and alpha, omega -dicarboxylic acid synthesis in root and seed suberin polyester. Plant Physiol. 150, 1831–1843. doi: 10.1104/pp.109.141408 19525321 PMC2719127

[B15] Delude C. Fouillen L. Bhar P. Cardinal M.-J. Pascal S. Santos P.. (2016). Primary fatty alcohols are major components of suberized root tissues of arabidopsis in the form of alkyl hydroxycinnamates. Plant Physiol. 171, 1934–1950. doi: 10.1104/pp.16.00834 27231100 PMC4936593

[B16] Delude C. Vishwanath S. J. Rowland O. Domergue F. (2017). Root aliphatic suberin analysis using non-extraction or solvent-extraction methods. Bio-Protoc 7, e2331. doi: 10.21769/BioProtoc.2331 34541091 PMC8410265

[B17] Domergue F. Kosma D. K. (2017). Occurrence and biosynthesis of alkyl hydroxycinnamates in plant lipid barriers. Plants 6, 25. doi: 10.3390/plants6030025 28665304 PMC5620581

[B18] Domergue F. Vishwanath S. J. Joubès J. Ono J. Lee J. A. Bourdon M.. (2010). Three arabidopsis fatty acyl-coenzyme a reductases, FAR1, FAR4, and FAR5, generate primary fatty alcohols associated with suberin deposition. Plant Physiol. 153, 1539–1554. doi: 10.1104/pp.110.158238 20571114 PMC2923872

[B19] Earley K. W. Haag J. R. Pontes O. Opper K. Juehne T. Song K.. (2006). Gateway-compatible vectors for plant functional genomics and proteomics. Plant J. Cell Mol. Biol. 45, 616–629. doi: 10.1111/j.1365-313X.2005.02617.x 16441352

[B20] Falginella L. Andre C. M. Legay S. Lin-Wang K. Dare A. P. Deng C.. (2021). Differential regulation of triterpene biosynthesis induced by an early failure in cuticle formation in apple. Hortic. Res. 8, 1–15. doi: 10.1038/s41438-021-00511-4 33790248 PMC8012369

[B21] Faust M. Shear C. B. (1972). Russeting of apples, an interpretive review. Hortscience 7, 233–235. doi: 10.21273/HORTSCI.7.3.233

[B22] Fernández-Pérez F. Pomar F. Pedreño M. A. Novo-Uzal E. (2015). Suppression of arabidopsis peroxidase 72 alters cell wall and phenylpropanoid metabolism. Plant Sci. Int. J. Exp. Plant Biol. 239, 192–199. doi: 10.1016/j.plantsci.2015.08.001 26398803

[B23] Franke R. Höfer R. Briesen I. Emsermann M. Efremova N. Yephremov A.. (2009). The DAISY gene from arabidopsis encodes a fatty acid elongase condensing enzyme involved in the biosynthesis of aliphatic suberin in roots and the chalaza-micropyle region of seeds. Plant J. Cell Mol. Biol. 57, 80–95. doi: 10.1111/j.1365-313X.2008.03674.x 18786002

[B24] Fraser C. M. Chapple C. (2011). The phenylpropanoid pathway in arabidopsis. Arab. Book 9, e0152. doi: 10.1199/tab.0152 PMC3268504 22303276

[B25] Gasic K. Hernandez A. Korban S. S. (2004). RNA Extraction from different apple tissues rich in polyphenols and polysaccharides for cDNA library construction. Plant Mol. Biol. Rep. 22, 437–438. doi: 10.1007/BF02772687

[B26] Graça J. (2015). Suberin: the biopolyester at the frontier of plants. Front. Chem. 3. doi: 10.3389/fchem.2015.00062 PMC4626755 26579510

[B27] Guerriero G. Achen C. Xu X. Planchon S. Leclercq C. C. Sergeant K.. (2021). The cell wall proteome of craterostigma plantagineum cell cultures habituated to dichlobenil and isoxaben. Cells 10, 2295. doi: 10.3390/cells10092295 34571944 PMC8468770

[B28] Höfer R. Briesen I. Beck M. Pinot F. Schreiber L. Franke R. (2008). The arabidopsis cytochrome P450 CYP86A1 encodes a fatty acid omega-hydroxylase involved in suberin monomer biosynthesis. J. Exp. Bot. 59, 2347–2360. doi: 10.1093/jxb/ern101 18544608 PMC2423664

[B29] Jensen J. K. Sørensen S. O. Harholt J. Geshi N. Sakuragi Y. Møller I.. (2008). Identification of a xylogalacturonan xylosyltransferase involved in pectin biosynthesis in arabidopsis. Plant Cell 20, 1289–1302. doi: 10.1105/tpc.107.050906 18460606 PMC2438468

[B30] Jin H. Cominelli E. Bailey P. Parr A. Mehrtens F. Jones J.. (2000). Transcriptional repression by AtMYB4 controls production of UV-protecting sunscreens in arabidopsis. EMBO J. 19, 6150–6161. doi: 10.1093/emboj/19.22.6150 11080161 PMC305818

[B31] Kamiya T. Borghi M. Wang P. Danku J. M. C. Kalmbach L. Hosmani P. S.. (2015). The MYB36 transcription factor orchestrates casparian strip formation. Proc. Natl. Acad. Sci. 112, 10533–10538. doi: 10.1073/pnas.1507691112 26124109 PMC4547244

[B32] Kashyap A. Jiménez-Jiménez Á.L. Zhang W. Capellades M. Srinivasan S. Laromaine A.. (2022). Induced ligno-suberin vascular coating and tyramine-derived hydroxycinnamic acid amides restrict ralstonia solanacearum colonization in resistant tomato. New Phytol. 234, 1411–1429. doi: 10.1111/nph.17982 35152435

[B33] Khanal B. P. Grimm E. Knoche M. (2012). Russeting in apple and pear: a plastic periderm replaces a stiff cuticle. AoB Plants 5, 1–12. doi: 10.1093/aobpla/pls048 PMC3553398 23350024

[B34] Knoche M. Khanal B. P. Stopar M. (2011). Russeting and microcracking of ‘Golden delicious’ apple fruit concomitantly decline due to gibberellin A4+7 application. J. Am. Soc Hortic. Sci. 136, 159–164. doi: 10.21273/JASHS.136.3.159

[B35] Kosma D. K. Murmu J. Razeq F. M. Santos P. Bourgault R. Molina I.. (2014). AtMYB41 activates ectopic suberin synthesis and assembly in multiple plant species and cell types. Plant J. Cell Mol. Biol. 80, 216–229. doi: 10.1111/tpj.12624 PMC4321041 25060192

[B36] Landgraf R. Smolka U. Altmann S. Eschen-Lippold L. Senning M. Sonnewald S.. (2014). The ABC transporter ABCG1 is required for suberin formation in potato tuber periderm. Plant Cell 26, 3403–3415. doi: 10.1105/tpc.114.124776 25122151 PMC4371835

[B37] Lashbrooke J. Aharoni A. Costa F. (2015). Genome investigation suggests MdSHN3, an APETALA2-domain transcription factor gene, to be a positive regulator of apple fruit cuticle formation and an inhibitor of russet development. J. Exp. Bot. 66, 6579–6589. doi: 10.1093/jxb/erv366 26220084 PMC4623677

[B38] Lashbrooke J. Cohen H. Levy-Samocha D. Tzfadia O. Panizel I. Zeisler V.. (2016). MYB107 and MYB9 homologs regulate suberin deposition in angiosperms. Plant Cell 28, 2097–2116. doi: 10.1105/tpc.16.00490 27604696 PMC5059810

[B39] Lee S.-B. Jung S.-J. Go Y.-S. Kim H.-U. Kim J.-K. Cho H.-J.. (2009). Two arabidopsis 3-ketoacyl CoA synthase genes, KCS20 and KCS2/DAISY, are functionally redundant in cuticular wax and root suberin biosynthesis, but differentially controlled by osmotic stress. Plant J. Cell Mol. Biol. 60, 462–475. doi: 10.1111/j.1365-313X.2009.03973.x 19619160

[B40] Legay S. Cocco E. André C. M. Guignard C. Hausman J.-F. Guerriero G. (2017). Differential lipid composition and gene expression in the semi-russeted “Cox orange pippin” apple variety. Front. Plant Sci. 8. doi: 10.3389/fpls.2017.01656 PMC5623121 29018466

[B41] Legay S. Guerriero G. André C. Guignard C. Cocco E. Charton S.. (2016). MdMyb93 is a regulator of suberin deposition in russeted apple fruit skins. New Phytol. 212, 977–991. doi: 10.1111/nph.14170 27716944

[B42] Legay S. Guerriero G. Deleruelle A. Lateur M. Evers D. André C. M.. (2015). Apple russeting as seen through the RNA-seq lens: strong alterations in the exocarp cell wall. Plant Mol. Biol. 88, 21–40. doi: 10.1007/s11103-015-0303-4 25786603

[B43] Le Mauff F. Loutelier-Bourhis C. Bardor M. Berard C. Doucet A. D’Aoust M.. (2017). Cell wall biochemical alterations during agrobacterium-mediated expression of haemagglutinin-based influenza virus-like vaccine particles in tobacco. Plant Biotechnol. J. 15, 285–296. doi: 10.1111/pbi.12607 27483398 PMC5316917

[B44] Li F. Ren S. Zhang W. Xu Z. Xie G. Chen Y.. (2013). Arabinose substitution degree in xylan positively affects lignocellulose enzymatic digestibility after various NaOH/H2SO4 pretreatments in miscanthus. Bioresour. Technol. 130, 629–637. doi: 10.1016/j.biortech.2012.12.107 23334020

[B45] Lima R. B. dos Santos T. B. Vieira L. G. E. Ferrarese M. de L. L. Ferrarese-Filho O.. (2013). Heat stress causes alterations in the cell-wall polymers and anatomy of coffee leaves (Coffea arabica l.). Carbohydr. Polym. 93, 135–143. doi: 10.1016/j.carbpol.2012.05.015 23465912

[B46] Ma F. Peterson C. A. (2000). Plasmodesmata in onion (Alliurn cepa l.) roots: a study enabled by improved fixation and embedding techniques. Protoplasma 211, 103–115. doi: 10.1007/BF01279903

[B47] McCartney L. Marcus S. E. Knox J. P. (2005). Monoclonal antibodies to plant cell wall xylans and arabinoxylans. J. Histochem. Cytochem. 53, 543–546. doi: 10.1369/jhc.4B6578.2005 15805428

[B48] Mishler-Elmore J. W. Zhou Y. Sukul A. Oblak M. Tan L. Faik A.. (2021)Extensins: Self-assembly, crosslinking, and the role of peroxidases (Accessed October 6, 2022). 10.3389/fpls.2021.664738 PMC8160292 34054905

[B49] Molina I. Kosma D. (2015). Role of HXXXD-motif/BAHD acyltransferases in the biosynthesis of extracellular lipids. Plant Cell Rep. 34, 587–601. doi: 10.1007/s00299-014-1721-5 25510356

[B50] Molina I. Li-Beisson Y. Beisson F. Ohlrogge J. B. Pollard M. (2009). Identification of an arabidopsis feruloyl-coenzyme a transferase required for suberin synthesis. Plant Physiol. 151, 1317–1328. doi: 10.1104/pp.109.144907 19759341 PMC2773081

[B51] Mortazavi A. Williams B. A. McCue K. Schaeffer L. Wold B. (2008). Mapping and quantifying mammalian transcriptomes by RNA-seq. Nat. Methods 5, 621–628. doi: 10.1038/nmeth.1226 18516045 PMC13303166

[B52] Nakasugi K. Crowhurst R. Bally J. Waterhouse P. (2014). Combining transcriptome assemblies from multiple *De novo* assemblers in the allo-tetraploid plant nicotiana benthamiana. PloS One 9, e91776. doi: 10.1371/journal.pone.0091776 24614631 PMC3948916

[B53] Nishimura H. Kamiya A. Nagata T. Katahira M. Watanabe T. (2018). Direct evidence for α ether linkage between lignin and carbohydrates in wood cell walls. Sci. Rep. 8, 6538. doi: 10.1038/s41598-018-24328-9 29695732 PMC5916878

[B54] Nomberg G. Marinov O. Arya G. C. Manasherova E. Cohen H. (2022). The key enzymes in the suberin biosynthetic pathway in plants: An update. Plants 11, 392. doi: 10.3390/plants11030392 35161373 PMC8839845

[B55] Panikashvili D. Shi J. X. Bocobza S. Franke R. B. Schreiber L. Aharoni A. (2010). The arabidopsis DSO/ABCG11 transporter affects cutin metabolism in reproductive organs and suberin in roots. Mol. Plant 3, 563–575. doi: 10.1093/mp/ssp103 20035035

[B56] Park S. Szumlanski A. L. Gu F. Guo F. Nielsen E. (2011). A role for CSLD3 during cell-wall synthesis in apical plasma membranes of tip-growing root-hair cells. Nat. Cell Biol. 13, 973–980. doi: 10.1038/ncb2294 21765420

[B57] Petit J. Bres C. Mauxion J.-P. Tai F. W. J. Martin L. B. B. Fich E. A.. (2016). The glycerol-3-Phosphate acyltransferase GPAT6 from tomato plays a central role in fruit cutin biosynthesis. Plant Physiol. 171, 894–913. doi: 10.1104/pp.16.00409 27208295 PMC4902622

[B58] Raudvere U. Kolberg L. Kuzmin I. Arak T. Adler P. Peterson H. (2019). g:Profiler: a web server for functional enrichment analysis and conversions of gene lists. Nucleic Acids Res 47(W1), W191–W198. doi: 10.1093/nar/gkz369 31066453 PMC6602461

[B59] Rennie E. A. Hansen S. F. Baidoo E. E. K. Hadi M. Z. Keasling J. D. Scheller H. V. (2012). Three members of the arabidopsis glycosyltransferase family 8 are xylan Glucuronosyltransferases1[W][OA]. Plant Physiol. 159, 1408–1417. doi: 10.1104/pp.112.200964 22706449 PMC3428776

[B60] Scheller H. V. Ulvskov P. (2010). Hemicelluloses. Annu. Rev. Plant Biol. 61, 263–289. doi: 10.1146/annurev-arplant-042809-112315 20192742

[B61] Schröder R. Atkinson R. G. Redgwell R. J. (2009). Re-interpreting the role of endo-β-mannanases as mannan endotransglycosylase/hydrolases in the plant cell wall. Ann. Bot. 104, 197–204. doi: 10.1093/aob/mcp120 19454593 PMC2710900

[B62] Serra O. Geldner N. (2022). The making of suberin. New Phytol. 235, 848–866. doi: 10.1111/nph.18202 35510799 PMC9994434

[B63] Shukla V. Han J.-P. Cléard F. Lefebvre-Legendre L. Gully K. Flis P.. (2021). Suberin plasticity to developmental and exogenous cues is regulated by a set of MYB transcription factors. Proc. Natl. Acad. Sci. U. S. A. 118, e2101730118. doi: 10.1073/pnas.2101730118 34551972 PMC8488582

[B64] Skene D. S. (1981). Wound healing in apple fruits: The anatomical response of cox’s orange pippin at different stages of development. J. Hortic. Sci. 56, 145–153. doi: 10.1080/00221589.1981.11514980

[B65] To A. Joubès J. Thueux J. Kazaz S. Lepiniec L. Baud S. (2020). AtMYB92 enhances fatty acid synthesis and suberin deposition in leaves of nicotiana benthamiana. Plant J. Cell Mol. Biol. 103, 660–676. doi: 10.1111/tpj.14759 32246506

[B66] Traore S. M. Zhao B. (2011). A novel gateway®-compatible binary vector allows direct selection of recombinant clones in agrobacterium tumefaciens. Plant Methods 7, 42. doi: 10.1186/1746-4811-7-42 22145613 PMC3265438

[B67] Ursache R. De Jesus Vieira Teixeira C. Dénervaud Tendon V. Gully K. De Bellis D. Schmid-Siegert E.. (2021). GDSL-domain proteins have key roles in suberin polymerization and degradation. Nat. Plants 7, 353–364. doi: 10.1038/s41477-021-00862-9 33686223 PMC7610369

[B68] Vishwanath S. J. Delude C. Domergue F. Rowland O. (2015). Suberin: biosynthesis, regulation, and polymer assembly of a protective extracellular barrier. Plant Cell Rep. 34, 573–586. doi: 10.1007/s00299-014-1727-z 25504271

[B69] Wellesen K. Durst F. Pinot F. Benveniste I. Nettesheim K. Wisman E.. (2001). Functional analysis of the LACERATA gene of arabidopsis provides evidence for different roles of fatty acid omega -hydroxylation in development. Proc. Natl. Acad. Sci. U. S. A. 98, 9694–9699. doi: 10.1073/pnas.171285998 11493698 PMC55514

[B70] Woolfson K. N. Esfandiari M. Bernards M. A. (2022). Suberin biosynthesis, assembly, and regulation. Plants 11, 555. doi: 10.3390/plants11040555 35214889 PMC8875741

[B71] Woolfson K. N. Haggitt M. L. Zhang Y. Kachura A. Bjelica A. Rey Rincon M. A.. (2018). Differential induction of polar and non-polar metabolism during wound-induced suberization in potato (Solanum tuberosum l.) tubers. Plant J. Cell Mol. Biol. 93, 931–942. doi: 10.1111/tpj.13820 29315972

[B72] Wu A.-M. Hörnblad E. Voxeur A. Gerber L. Rihouey C. Lerouge P.. (2010). Analysis of the arabidopsis IRX9/IRX9-l and IRX14/IRX14-l pairs of glycosyltransferase genes reveals critical contributions to biosynthesis of the hemicellulose glucuronoxylan. Plant Physiol. 153, 542–554. doi: 10.1104/pp.110.154971 20424005 PMC2879767

[B73] Xie B. Wang X. Zhu M. Zhang Z. Hong Z. (2011). CalS7 encodes a callose synthase responsible for callose deposition in the phloem. Plant J. 65, 1–14. doi: 10.1111/j.1365-313X.2010.04399.x 21175885

[B74] Xu X. Backes A. Legay S. Berni R. Faleri C. Gatti E.. (2019). Cell wall composition and transcriptomics in stem tissues of stinging nettle (Urtica dioica l.): Spotlight on a neglected fibre crop. Plant Direct 3, e00151. doi: 10.1002/pld3.151 31417976 PMC6689792

[B75] Xu X. Guerriero G. Berni R. Sergeant K. Guignard C. Lenouvel A.. (2022b)MdMYB52 regulates lignin biosynthesis upon the suberization process in apple (Accessed October 19, 2022). 10.3389/fpls.2022.1039014 PMC9583409 36275517

[B76] Xu H. Liu P. Wang C. Wu S. Dong C. Lin Q.. (2022a). Transcriptional networks regulating suberin and lignin in endodermis link development and ABA response. Plant Physiol. 190, 1165–1181. doi: 10.1093/plphys/kiac298 35781829 PMC9516719

[B77] Yadav V. Molina I. Ranathunge K. Castillo I. Q. Rothstein S. J. Reed J. W. (2014). ABCG transporters are required for suberin and pollen wall extracellular barriers in arabidopsis. Plant Cell 26, 3569–3588. doi: 10.1105/tpc.114.129049 25217507 PMC4213157

[B78] Yang J. Bak G. Burgin T. Barnes W. J. Mayes H. B. Peña M. J.. (2020). Biochemical and genetic analysis identify CSLD3 as a beta-1,4-Glucan synthase that functions during plant cell wall synthesis. Plant Cell 32, 1749–1767. doi: 10.1105/tpc.19.00637 32169960 PMC7203914

[B79] Yeats T. Vellosillo T. Sorek N. Ibáñez A. B. Bauer S. (2016). Rapid determination of cellulose, neutral sugars, and uronic acids from plant cell walls by one-step two-step hydrolysis and HPAEC-PAD. Bio-Protoc 6, e1978–e1978. doi: 10.21769/BioProtoc.1978

[B80] Yim K.-O. Bradford K. J. (1998). Callose deposition is responsible for apoplastic semipermeability of the endosperm envelope of muskmelon seeds. Plant Physiol. 118, 83. doi: 10.1104/pp.118.1.83 9733528 PMC34876

[B81] Zhao H. Kosma D. K. Lü S. (2021). Functional role of long-chain acyl-CoA synthetases in plant development and stress responses. Front. Plant Sci. 12. doi: 10.3389/fpls.2021.640996 PMC8019973 33828572

[B82] Zykwinska A. W. Ralet M.-C. J. Garnier C. D. Thibault J.-F. J. (2005). Evidence for *In vitro* binding of pectin side chains to cellulose. Plant Physiol. 139, 397–407. doi: 10.1104/pp.105.065912 16126855 PMC1203388

