# Supplementary figures and images for: Characterization of MdMYB68, a suberin master regulator in russeted apples
Source: Front Plant Sci. 2023 Mar 20;14:1143961. doi: 10.3389/fpls.2023.1143961 (PMC10067606; doi:10.3389/fpls.2023.1143961)

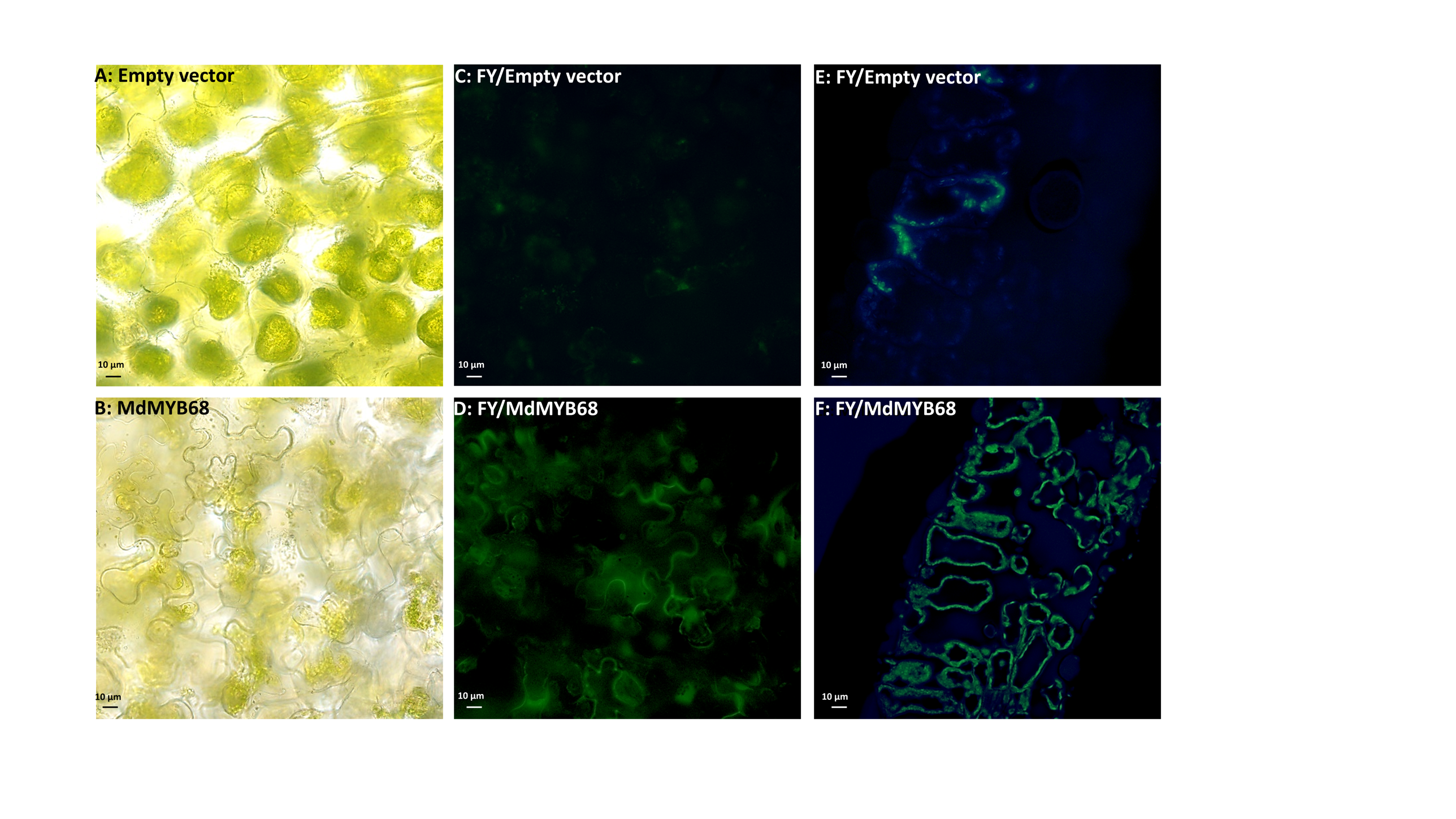

Supplement: Supplementary file 2 [file Image_2.tif]

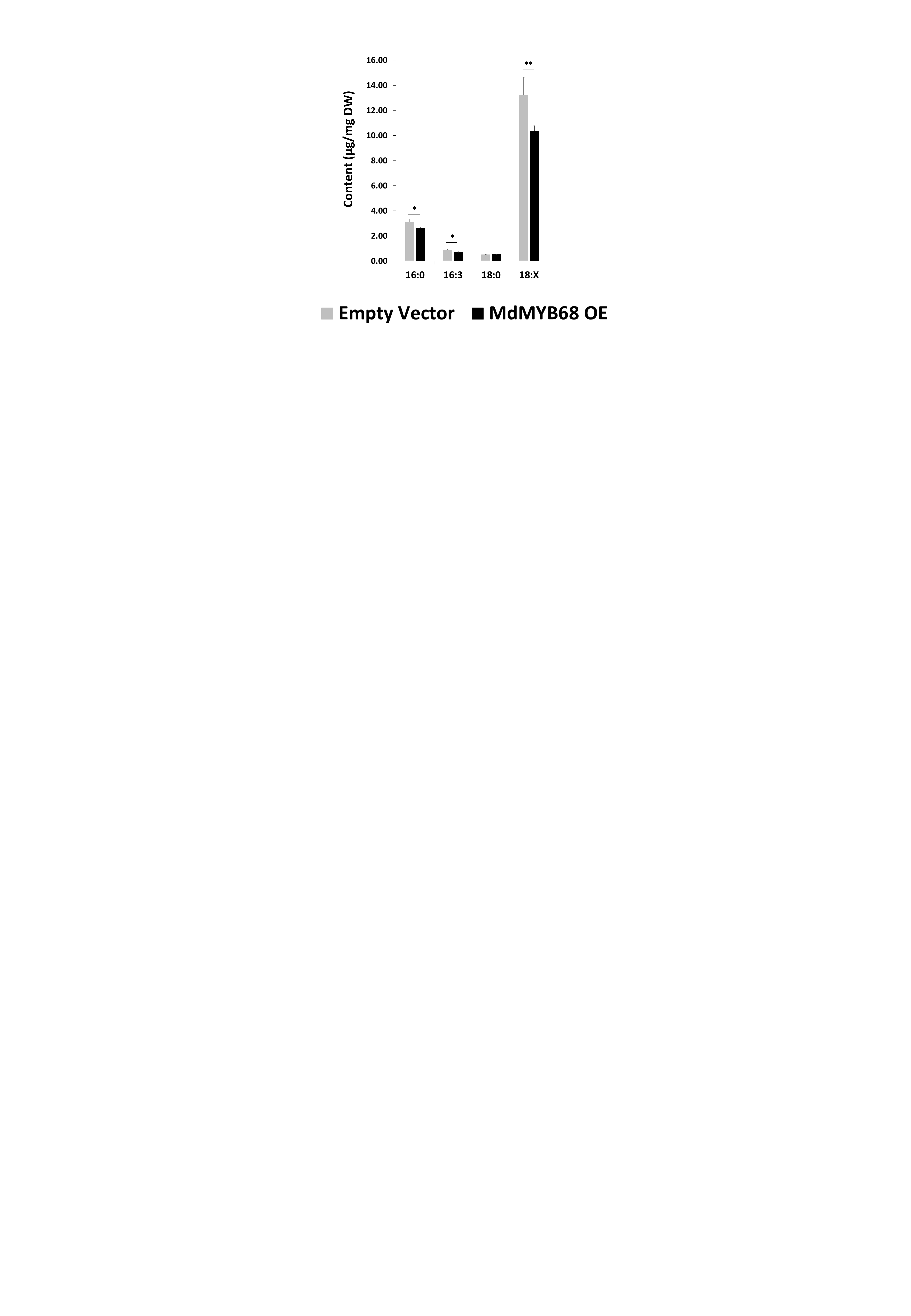

Supplement: Supplementary file 3 [file Image_3.tif]
