# Supplementary material for: Characterization of MdMYB68, a suberin master regulator in russeted apples
Source: Front Plant Sci. 2023 Mar 20;14:1143961. doi: 10.3389/fpls.2023.1143961 (PMC10067606; doi:10.3389/fpls.2023.1143961)
Supplement: Supplementary file 4 [file Image_4.pdf]

LM1

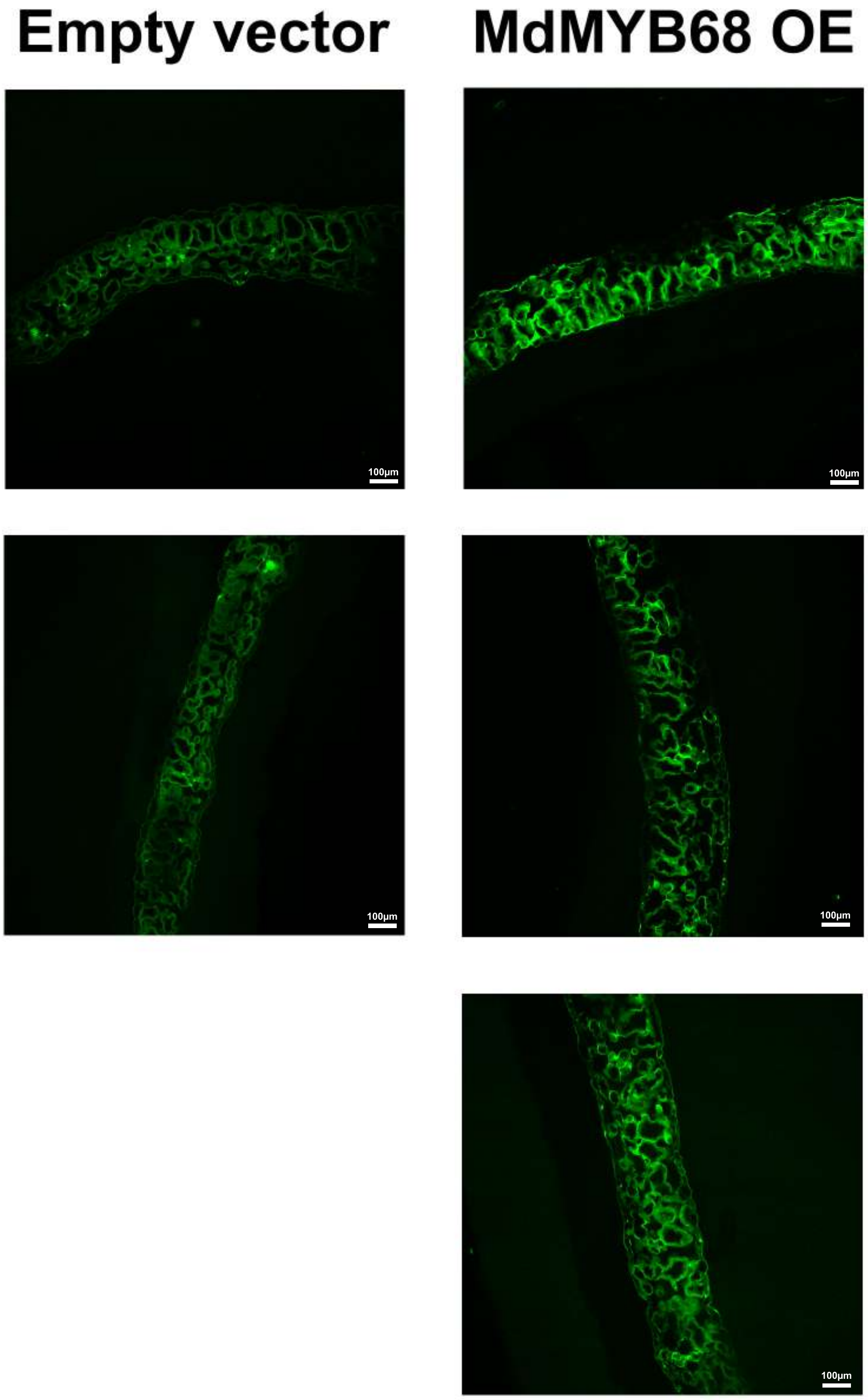

LM2

Empty vector MdMYB68 OE

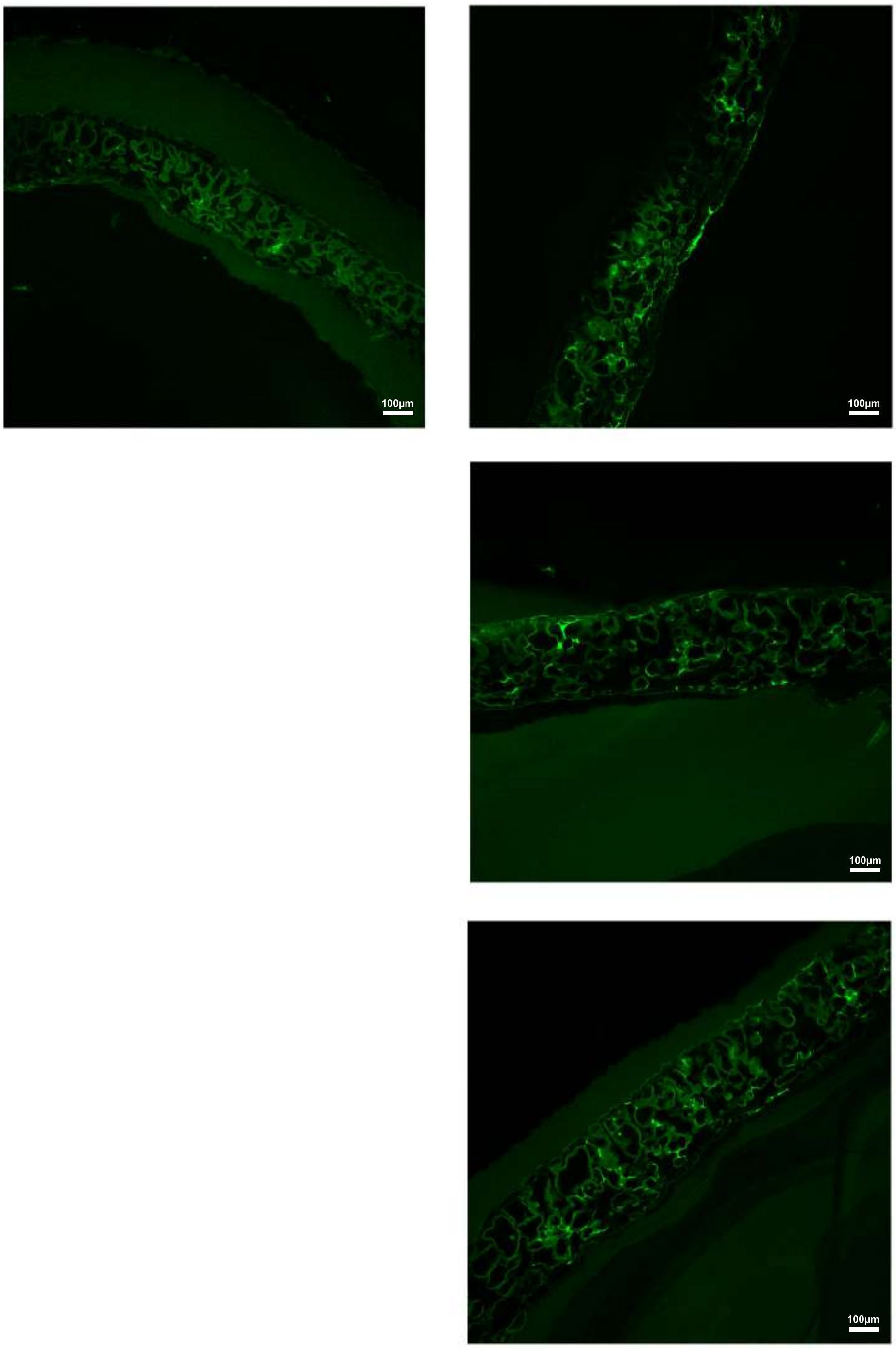

LM5

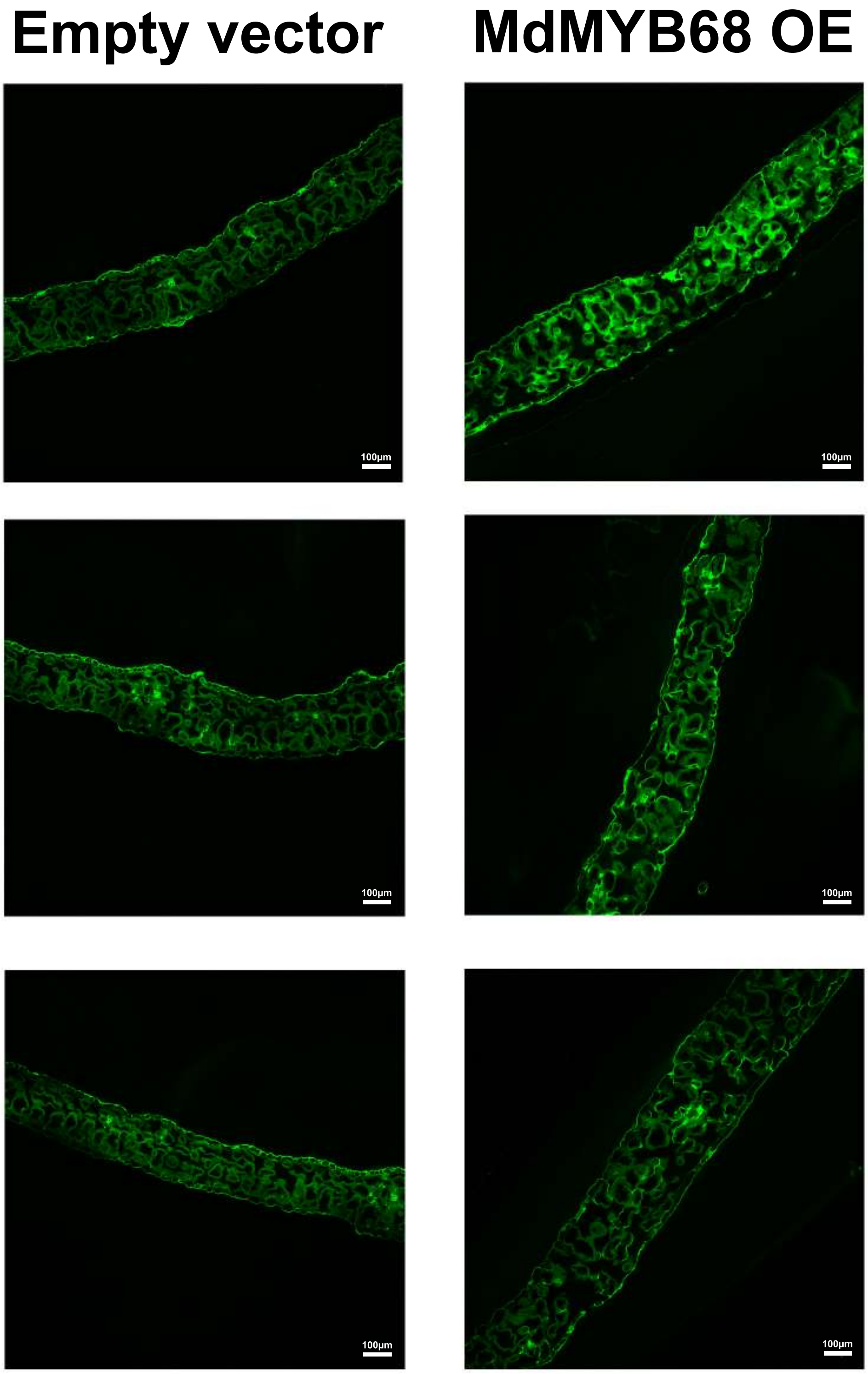

Empty vector
MdMYB68 OE
100μm
100μm
100μm
100μm
100μm
100μm

LM10

Empty vector **MdMYB68 OE**

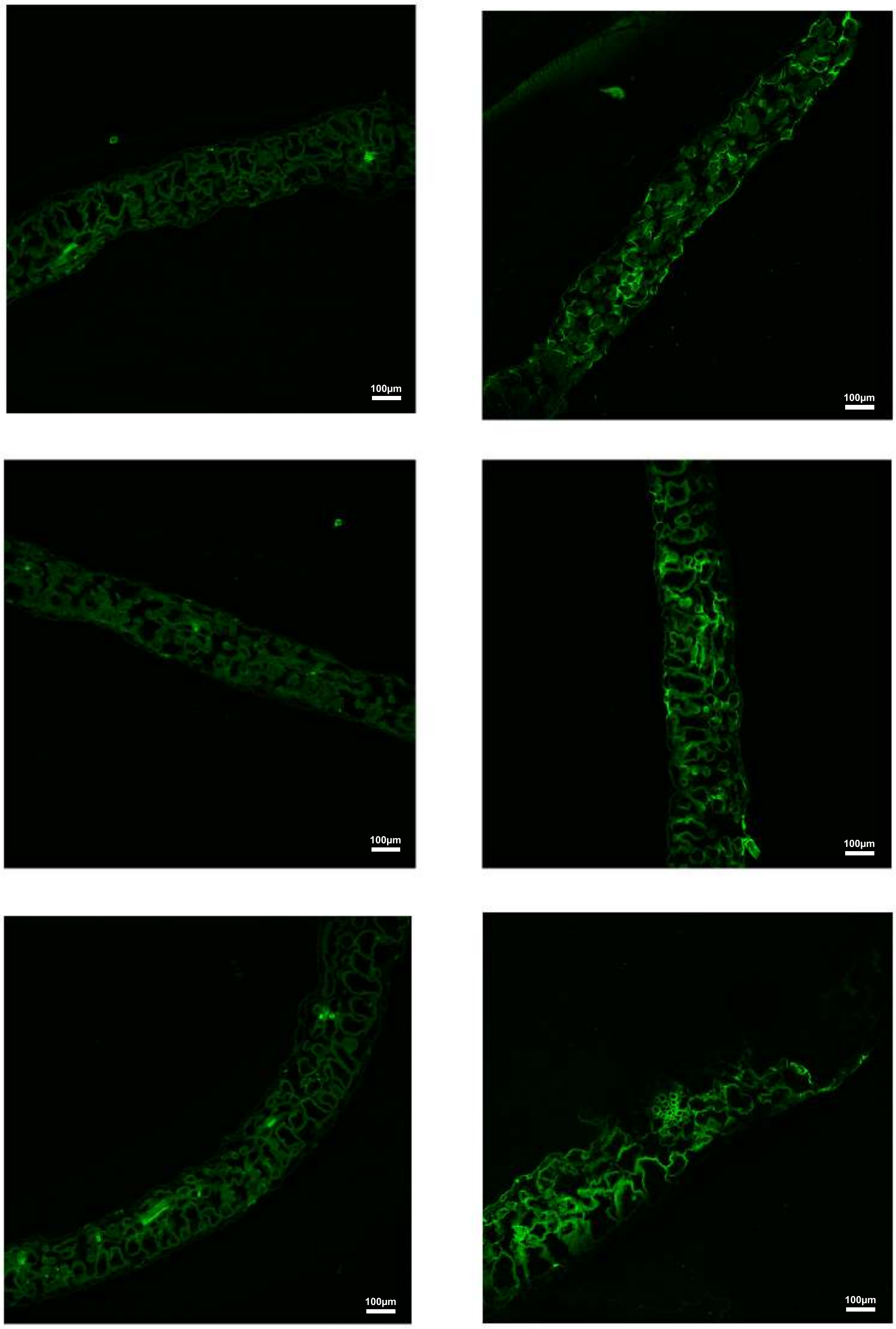

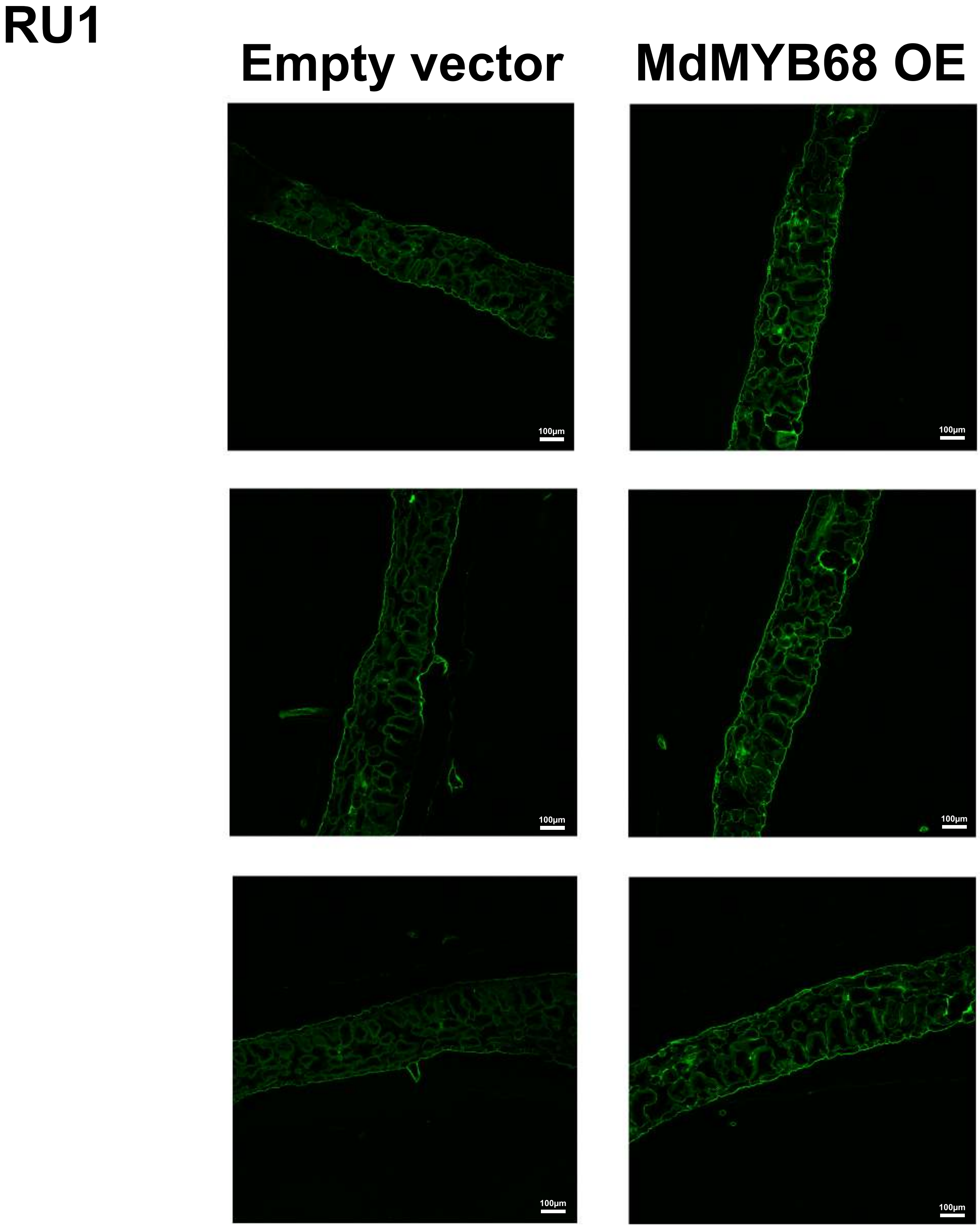
RU1
Empty vector
MdMYB68 OE
100µm
100µm
100µm
100µm
100µm
100µm
