# Supplementary material for: Characterization of MdMYB68, a suberin master regulator in russeted apples
Source: Front Plant Sci. 2023 Mar 20;14:1143961. doi: 10.3389/fpls.2023.1143961 (PMC10067606; doi:10.3389/fpls.2023.1143961)
Supplement: Supplementary file 10 [file Table_6.docx]

Supplementary figure 1: Gene interaction matrix obtained from genes significantly upregulated in russeted apple fruit skin compared to waxy apples (-3<log2 ratio (russeted/waxy)<3) (Legay et al., 2015; André et al., 2022). Grey lines represent the gene interaction confidence (ranged from 0 to 1): thickest lines display confidence scores higher than 0.9, thinnest lines display confidence scores between 0.4 and 0.7.

Supplementary figure 2: Optical microscope pictures showing non-included (A, B, C, D) and resin-included (E, F) tobacco leaves. The leaves were collected 7 days after infiltration with MdMYB68 and empty vector and stained using Fluorol Yellow 088. (A, B) Adaxial side of leaves observed in bright field. (C, D) Adaxial side of leaves exposed to UV light and observed through a DAPI filter. (E, F) Resin-included leave sections exposed to UV light and observed through a DAPI filter.

Supplementary figure 3: C16 to C18 fatty acid contents obtained from the soluble lipid fraction obtained from *Nicotiana benthamiana* leaves collected 7 days after agroinfiltration with the MdMYB68 construct (MdMYB68 OE) or the Control/empty vector construct (Empty vector) using GC-MS analysis. 4 biological replicates were used (n=4). Values are expressed as the mean ± SD from four biological replicates. One, two and three asterisks indicate significant differences at p< 0.05, p<0.01, p<0.001.
